# Endomembrane Protein Trafficking Regulated by a *Tv*CyP2 Cyclophilin in the Protozoan Parasite, *Trichomonas vaginalis*

**DOI:** 10.1038/s41598-020-58270-6

**Published:** 2020-01-27

**Authors:** Hong-Ming Hsu, Yu-Hsin Huang, Sarita Aryal, Hsing-Wei Liu, Chinpan Chen, Shu-Hui Chen, Chien-Hsin Chu, Jung-Hsiang Tai

**Affiliations:** 1 Department of Tropical Medicine and Parasitology, College of Medicine, National Taiwan University, Taipei, Taiwan; 2 Division of Infectious Diseases and Immunology, Institute of Biomedical Sciences, Academia Sinica Taipei, Taiwan; 3 Structural Biology, Institute of Biomedical Sciences, Academia Sinica Taipei, Taiwan; 4 Department of Chemistry, National Cheng Kung University, Tainan, Taiwan

**Keywords:** Endoplasmic reticulum, Protein translocation

## Abstract

In *Trichomonas vaginalis*, the *Tv*CyP1-catalyzed conformational switches of two glycinyl-prolyl imide bonds in Myb3 were previously shown to regulate the trafficking of Myb3 from cytoplasmic membrane compartments towards the nucleus. In this study, *Tv*CyP2 was identified as a second cyclophilin that binds to Myb3 at the same dipeptide motifs. The enzymatic proficiency of *Tv*CyP2, but not its binding to Myb3, was aborted by a mutation of Arg^75^ in the catalytic domain. *Tv*CyP2 was localized to the endoplasmic reticulum with a weak signal that extensively extends into the cytoplasm as well as to the plasma membrane according to an immunofluorescence assay. Moreover, *Tv*CyP2 was co-enriched with *Tv*CyP1 and Myb3 in various membrane fractions purified by differential and gradient centrifugation. *Tv*CyP2 was found to proficiently enzymatically regulate the distribution of *Tv*CyP1 and Myb3 among purified membrane fractions, and to localize *Tv*CyP1 in hydrogenosomes and on plasma membranes. Protein complexes immunoprecipitated from lysates of cells overexpressing *Tv*CyP1 and *Tv*CyP2 were found to share some common components, like *Tv*CyP1, *Tv*CyP2, *Tv*Bip, Myb3, *Tv*HSP72, and the hydrogenosomal heat shock protein 70 (HSP70). Direct interaction between *Tv*CyP1 and *Tv*CyP2 was confirmed by a GST pull-down assay. Fusion of vesicles with hydrogenosomes was observed by transmission electron microscopy, whereas *Tv*CyP1, *Tv*CyP2, and Myb3 were each detected at the fusion junction by immunoelectron microscopy. These observations suggest that *T. vaginalis* may have evolved a novel protein trafficking pathway to deliver proteins among the endomembrane compartments, hydrogenosomes and plasma membranes.

## Introduction

*Trichomonas vaginalis* is a p arasitic protozoan that survives only as trophozoites in the human urogenital tract^1^. Trichomoniasis has long held the position as the most common sexually transmitted disease of nonviral origin^2^. The infection often manifests mild symptoms or is asymptomatic, but it can sometimes cause adverse outcomes during pregnancy, ranging from preterm deliveries or low birth weights to abortions and stillbirths^3,4^. Trichomoniasis is also recognized as a risk factor for the elevated transmission of the human immunodeficiency virus (HIV) and papillomaviruses, and the development of progressive cervical and prostate cancers^5–8^. Trichomoniasis can be easily cured by metronidazole, a drug commonly used for gram-negative bacterial infections and luminal giardiasis and entamebiasis, but reports of drug-resistant clinical isolates have been increasing over the years^3^. The infection is often overlooked, and transmission of *T. vaginalis* is difficult to control^9^, rendering this neglected parasite an emerging threat to public health.

Among environmental factors, iron was shown to modulate the virulence of this parasite via its effects on the transcription of myriad genes^10–12^. Accordingly, iron was shown to regulate expression levels, protein trafficking, and promoter entry of three transcription factors, Myb1, Myb2, and Myb3, which in coordination may regulate overall expression of a malic enzyme gene, reputed to be *ap65-1*^13–15^. While ectopic expression of the malic enzyme and its role in the cytoadherence of the parasite remain controversial^1,16,17^, regulation of *ap65-1* transcription has provided a useful platform to study signal transduction and protein trafficking in this intriguing parasite^18,19^. Like other members of the eukaryotic Myb protein family, Myb1, Myb2, and Myb3 each harbors conserved R2R3 DNA-binding domains like human c-Myb^13–15,20^. Unlike c-Myb, which harbors a cluster of four to six positively charged amino acids as the nuclear localization signal^21^, nuclear import of Myb2 and Myb3 is each mediated by the entire R2R3 domain^22,23^. Myb1 and Myb3 are mostly associated with membranes^18,24^, yet their nuclear import requires conformational switches between the *cis* and *trans* interconversion of glycinyl-prolyly (Gly-Pro) imide bonds catalyzed by the cyclophilin-type peptidyl-prolyl isomerase, *Tv*CyP1^18,24^. As a homolog of human cyclophilin subtype CyPA (*h*CyPA)^25^, *Tv*CyP1 has a conserved catalytic domain, the enzymatic proficiency of which is aborted by binding to the commonly used immunosuppressive drug, cyclosporine A (CsA)^18^. *Tv*CyP1 can be detected in multiple membrane compartments; yet it resides primarily in hydrogenosomes^18,24^, implying that *Tv*CyP1 may be translocated from endomembrane compartments into hydrogenosomes. Such a membrane trafficking pathway is incongruent with our current understanding of the transport of hydrogenosomal proteins from the soluble cytosol into hydrogenosomes^18,24^.

In this report, *Tv*CyP2 was found to be a second cyclophilin, which mediates *cis-trans* interconversions of the two Gly-Pro bonds in Myb3. It was primarily localized to the endoplasmic reticulum (ER), but was found to regulate protein trafficking of *Tv*CyP1 and Myb3 towards hydrogenosomes and also *Tv*CyP1 towards plasma membranes. In addition to the biochemical evidence, fusion of some uncharacterized vesicles to hydrogenosomes was also observed, with *Tv*CyP2, *Tv*CyP1, and Myb3 identified at the fusion junction. These observations suggest that this parasite may harbor a novel membrane trafficking pathway to deliver proteins to hydrogenosomes and/or plasma membranes.

## Experimental Procedures

### Cultures

*Trichomonas vaginalis* T1 cells were maintained in TYI medium supplemented with 10% calf serum as previously described^26^. Cells with an initial density of 10^5^ cells ml^−1^ were grown to 1.5 × 10^6^ cells ml^−1^ overnight for the experiments.

### DNA transfection and selection of stable transfectants

Expression plasmids were electroporated into *T. vaginalis*, and stable cell lines were selected by paromomycin as previously described^26^.

### Oligonucleotides

Sequences of oligonucleotides used in the present study are listed in Table 1.

**Table 1:** Oligonucleotide primers used in the present study.

**Construct of HA-*Tv*CyP2 overexpression**	
*Tv*CyP2-BamHI-5′	AGGATCCATGTTAGCATTCTTTGCTAC
*Tv*CyP2-XhoI-3′	ACTCGAGTTACTCTGTGATTTCACCGC
**Mutation of** ***Tv*CyP2 (R75A)**	
*Tv*CyP2(R75A)-5′	TCTCCATTCCACGCAGTTATCCCTAACTTCATGATTC
*Tv*CyP2(R75A)-3′	AGGGATAACTGCGTGGAATGGAGAGCCCTTGTAGTG
**Construct of pET28a-*Tv*Arf-1**	
*Tv*Arf-1-BamHI-5′	AGGATCCATGGGTCTCTTATTCAGTGAAACATTC
*Tv*Arf-1-XhoI -3′	ACTCGAGTTAGAAGTCCTGGTTGATCAGATCACC

Restriction enzyme sites used in plasmid construction are underlined, and the sequences of nucleotides used for mutation are boxed.

### Construction of plasmids

**Figure 1:**
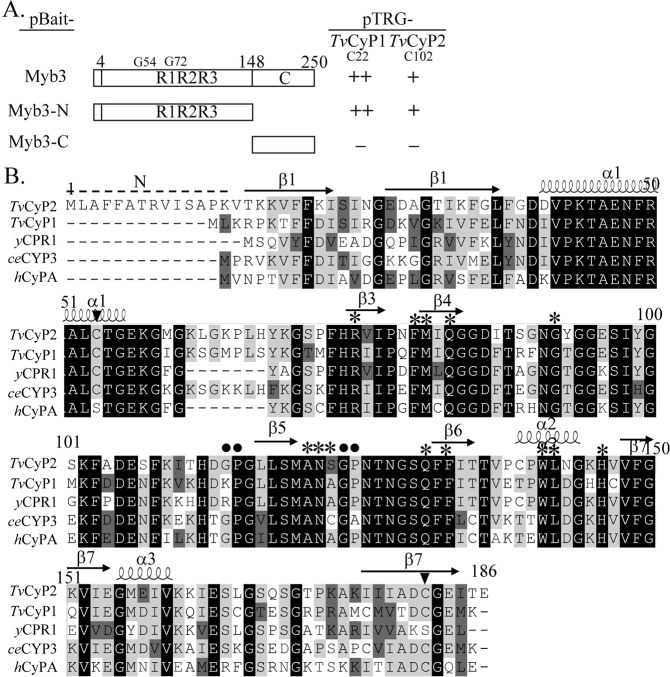
*Tv*CyP2 as a Myb3-binding protein. *Tv*CyP2 was identified as a Myb3-binding protein by two-hybrid library screening. In A, utilizing a pair-wise two-hybrid interaction assay, pBait-Myb3, pBait-Myb3-N, and pBait-Myb3-C were each paired with pTRG-c22 or pTRG-c102. The relative strength of the interaction as revealed by the formation of colonies in each assay (−, no colony formation; +, >30 colonies; ++, >100 colonies) is summarized in the right panel. In B, the sequence of *Tv*CyP2 (A2DLL4) was aligned to those of *Tv*CyP1 (A2DT06), *ce*CYP3 (P52011) in *Caenorhabditis elegans*, *y*CPR1 (P14832) in yeast, and *h*CyPA (P62937) in humans. The amino acids indicated by asterisks are involved in enzymatic proficiency and CsA binding. The Gly-Pro bond (closed circle) and conserved Cys (closed triangle) are also indicated.

To construct bait for library screening of a bacterial two-hybrid system (Stratagene), DNA fragments spanning various regions of the Myb3 coding sequence (see Fig. 1A) were amplified from pET28-Myb3^14^ by a polymerase chain reaction (PCR) using the primer pair, NotI-(x)-pBT-5′ and XhoI-(y)-pBT-3′ (x and y indicate the locations of the N- and C-terminal amino acids, respectively). The PCR product was gel-purified and cloned into pGEM-T Easy (Promega). The *Not*I- and *Xho*I-predigested insert was cloned into a *Not*I- and *Xho*I-restricted pBT backbone to produce pBait-Myb3, pBait-Myb3/N, or pBait-Myb3/C (Fig. 1A). pET28-Myb3, pET28-Myb3(G54A), pET28-Myb1(G72A), pET28-Myb3(G54A/G72A), and pFLP-ha-*Tv*CyP1 were obtained as described in a previous report^24^.

To construct the plasmid for production of recombinant *Tv*Arf-1, the coding sequence of the *tvarf-1* gene (gene accession no. TVAG_301220) was amplified from *T. vaginalis* genomic DNA by the primer pair, *Tv*ARF-1-BamHI-5′ and *Tv*Arf-1-XhoI-3′, for cloning into pGEM-T Easy. The *BamH*I/*Xho*I-restricted insert was subcloned into pET28a predigested with BamHI/XhoI to generate pET28a-*Tv*Arf-1.

To overexpress hemoagglutinin (HA)-tagged *Tv*CyP2, the *tvcyp2* coding sequence was amplified from genomic DNA by a PCR using the primer pair, FLP-*Tv*Cyp2-BamHI-5′ and FLP-*Tv*Cyp2-XhoI-3′. The gel-purified PCR product was cloned into pGEM-T Easy (Promega), and the insert restricted by *BamH*I/*Xho*I was ligated with *BamH*I/*Xho*I-restricted pFLP-ha-*Tv*CyP1 to generate pFLP-ha*-Tv*CyP2. The two-step PCR described above was employed to mutate Arg^75^ in *Tv*CyP2 to alanine. Briefly, from pFLP-ha*-Tv*CyP2, a 5′-DNA fragment was amplified using the primer pair, FLP-seq^18^ and *Tv*Cyp2(R75A)-3′, and a 3′-DNA fragment using the primer pair, *Tv*CyP2(R75A)-5′ and sp6. The gel-purified PCR products were mixed, denatured, and annealed for a second PCR using the primer pair, FLP-seq and sp6. The PCR product digested with *BamH*I/*Xho*I was cloned into *BamH*I/*Xho*I-restricted pFLP-ha-*Tv*CyP2 to generate pFLP-ha-*Tv*CyP2(R75A).

To produce glutathione-S-transferase (GST) fusion proteins, pFLP-ha-*Tv*CyP2 or pFLP-ha*-Tv*CyP2(R75A) was digested with *BamH*I and *Xho*I. The insert was cloned into *BamH*I/*Xho*I-restricted pGEX-2T (GE Healthcare) to respectively generate pGST-*Tv*CyP2 or pGST-*Tv*CyP2(R75A).

*Two-hybrid* further screened on a dual selective medium containing 5 mM 3-amino-1,2,4-triazole and streptomycin. A pair-wise interaction assay using pBait-Myb3 and each positive clone in the pTRG vector for two-hybrid selection was performed to confirm the interacting pair.

### The expression and purification of recombinant proteins

pET*-Tv*CyP2 was transformed into *E. coli* BL21 (DE3). pET28-Myb3, pET28-Myb3(G54A), pET28-Myb1(G72A), and pET28-Myb3 (G54A/G72A) were transformed into *E. coli* BL21-CodonPlus (DE3)-RIL. pGST-*Tv*CyP2 and pGST-*Tv*CyP2(R75A) were transformed into *E. coli* DH5α. A colony from each transformation was inoculated in LB broth containing 50 μg ml^−1^ ampicillin and incubated at 37 °C with constant shaking. Expression of His-tagged proteins was induced at OD_600_ reached 0.6 in the presence of 1 mM isopropyl-β-D-galactoside (IPTG) for 3 h at 30 °C and purified using a His-bound nickel column as described by the supplier (Novagen). GST-fusion proteins were produced and purified as described by the supplier (GE Healthcare).

### Western blotting

Protein samples were separated by sodium dodecylsulfate polyacrylamide gel electrophoresis (SDS-PAGE). Proteins were stained with Coomassie blue or transferred to polyvinylidene difluoride (PVDF) membranes (Millipore) by a semidry electro-blotter for Western blotting. Antibodies from commercial sources, including rabbit anti-acetyl histone H3K9 (3000×) (Upstate), a mouse monoclonal anti-HA antibody (5000×) (HA-7, Sigma), and a mouse monoclonal anti-α-tubulin antibody (10,000×) (DM1A, Sigma), were used as described by the suppliers. Malic enzyme (ME), pyruvate ferrodoxin oxidoreductase A (PFO), Myb1, Myb2, Myb3, *Tv*CyP1, *Tv*CyP2, *Tv*Myb3IPhmw, binding immunoglobulin protein (*Tv*Bip), hydrogenosomal heat shock protein 70 (HdHSP70), heat shock protein 72 (*Tv*HSP72), and *Tv*14-3-3 proteins were respectively detected using mouse monoclonal 12G4 (1000×) (a gift from John Alderete, Washington State University, Pullman, WA, USA)^27^, rabbit anti-PFO (10^4^×) (a gift from Dr. Rossana Arroyo, CINVESTA, Mexico City, Mexico)^28,29^, mouse anti-Myb1 (1000×)^14^, rabbit anti-Myb2 (4000×)^15^, rabbit anti-Myb3 (3000×)^13^, rat anti-*Tv*CyP1 (5000×)^18^, rat anti-*Tv*CyP2 (1000×), rat anti-*Tv*Myb3IPhmw (3000×)^24^, rabbit anti-*Tv*Bip (10^4^×), rabbit anti-HdHSP70 (10^4^×)^30^ (all gifts from Patricia J. Johnson, UCLA Molecular Biology Institute, Los Angeles, CA, USA), rat anti-*Tv*HSP72 (2 × 10^4^×), and rat anti-*Tv*14-3-3 (3000×)^24^. Signals on blots were detected by an enhanced chemiluminescence (ECL) system as described by the supplier (Thermo Scientific). The relative intensities of signals were quantified and analyzed by MetaMorph software (Molecular Devices).

### GST pull-down assay

GST or GST-fusion proteins were purified from the bacterial expression system by glutathione-conjugated beads in a TEN200 buffer system (1 mM EDTA, 200 mM NaCl, and 20 mM Tris-HCl, at pH 7.4) at 4 °C according to the supplier’s instruction (GE healthcare). The subsequent GST pull down assay was performed as described^31^. Briefly, 30 picomoles of GST and particular GST-fusion proteins immobilized on 20 μl glutathione conjugated beads were each reacted with 35 nM of a specific His-tagged protein in 1 ml TEN200 buffer at 4 °C overnight. The reaction products were washed for 10 min in a washing buffer (0.1% TritonX-100 in TEN200 buffer) three times. The reaction products were separated by SDS-PAGE in a 12% gel for Coomassie blue staining and Western blotting. In some experiments, the blots were also stained by Ponceau S to show protein input levels.

### Peptidylprolyl isomerase enzymatic proficiency assay

**Figure 2:**
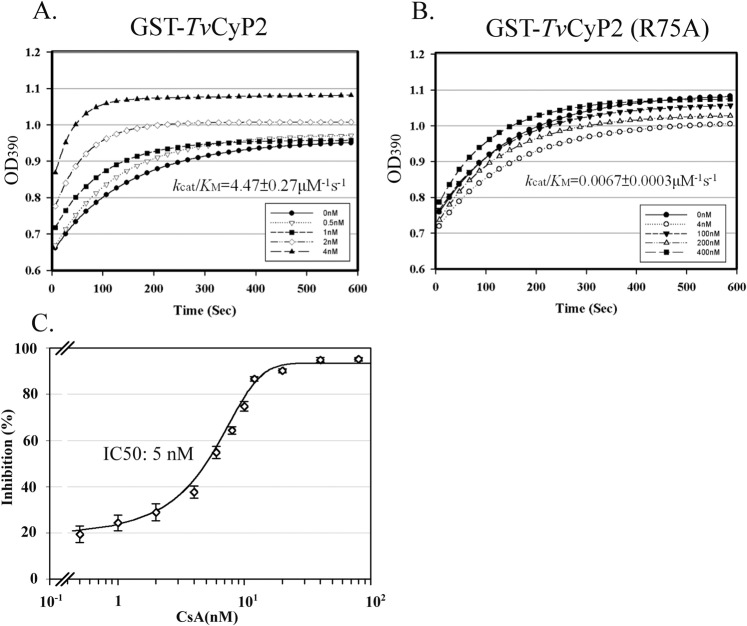
The enzymatic proficiency of *Tv*CyP2. In the enzymatic reaction, 0~4 nM of GST-*Tv*CyP2 (**A**) and GST-*Tv*CyP2(R75A) (**B**) were each reacted with a chromogenic substrate. The enzymatic reaction was measured at 1-s intervals over 10 min by monitoring the OD_390_ with a spectrophotometer. The logarithmic phase of the enzyme reaction is plotted against various concentrations of GST-*Tv*CyP2, and the calculated *k*_cat_/*K*_M_ value is listed below each panel The inhibitory effect of cyclosporine A (CsA) (0~80 nM) on the enzymatic reaction of 4 nM GST-*Tv*CyP2 is depicted in C.

The enzymatic proficiency of GST-*Tv*CyP2 or GST-*Tv*CyP2(R75A) was analyzed by measuring the *cis*-*trans* isomerization of the chromogenic peptide, N-succinyl-Ala-Ala-Pro-Phe-p-nitroanilide (Sigma), using a commonly used spectrophotometric method as previously described^32,33^. Briefly, 10 µl of the substrate (2.2 mM peptide in trifluorethanol and 0.45 M LiCl) and 30 μl of chymotrypsin (0.5 M) were mixed with 250 μl of 40 mM HEPES (pH 7.8) in a pre-chilled cuvette. The reaction was initiated at 10 °C and lasted for 10 min in a spectrophotometer (DU800, Beckman Coulter) with the addition of 10 μl of GST-*Tv*CyP2 diluted to the desired concentration. The OD_390_ value was recorded at 1-s intervals. Variations in the concentration of the added enzyme was used to produce a series of reaction curves as shown in Fig. 2A,B. The natural logarithms of differences between the OD_390_ and the final OD_390_ were plotted versus time over a span of 60 s to derive the first-order reaction constant (*k*_obs_). *k*_obs_ was then graphed against concentrations of the enzyme based on *k*_obs_ = *k*_cat_/*K*_M_ × [GST − *Tv*CyP2] to give the enzymatic proficiency *k*_cat_/*K*_M_^32,33^. To determine the 50% inhibitory concentration (IC_50_) of the inhibitor, 0~80 nM of cyclosporine A (CsA) serially diluted from a stock solution of 8.3 mM CsA in ethanol was added in the enzyme reaction containing 4 nM GST-*Tv*CyP2.

### Antibody production

His-*Tv*CyP2 and His-*Tv*Arf-1 were each used for immunization in mice and rats following a standard protocol^34^. Antisera were collected and purified by protein A-affinity chromatography as described by the supplier (Sigma).

### Immunofluorescence assay (IFA)

Cells were fixed in 4% paraformaldehyde in phosphate-buffered saline (PBS) for 15 min and permeated in 0.2% TritonX-100 in PBS for 15 min. In some experiments, fixed cells were not permeated to detect the proteins on a plasma membrane. The primary immunoreaction was performed using the mouse anti-HA (400×) (HA-7, Sigma), mouse anti-*Tv*CyP1 (1200×), rat anti-*Tv*CyP2 (400×), or rabbit anti-*Tv*Bip polyclonal antibody (gifts from Patricia J. Johnson, UCLA Molecular Biology Institute). Secondary immunoreactions were performed using FITC- or Cy3-conjugated secondary antibodies (Jackson Immunoresearch). Nuclei were stained with DAPI. Fluorescence signals were measured by confocal microscopy (LSM700 or LSM880, Zeiss). The intensities were quantified by MetaMorph as described by the supplier (Molecular Devices). Cell morphology was imaged by phase-contrast microscopy.

### Isothermal titration calorimetry (ITC)

ITC was performed at 25 °C using a MicroCal iTC200 system (Malvern, United Kingdom) for the protein-protein interactions. Briefly, His-*Tv*CyP1, His-*Tv*CyP2, and Myb3^53–180^, which was previously used for structural analysis of Myb3^35^, were purified, and kept in 20 mM phosphate buffer at pH 6.0 containing 50 mM NaCl. Two microliters of 1.5 mM Myb3^53–180^ was injected at intervals into 300 μl of 150 μM His-*Tv*CyP2 or His-*Tv*CyP1 in ITC cell. The data were collected and analyzed using the software Origin 7.0. The binding isotherms were fitted to the one-site binding model, giving values of the stoichiometry (n) of the interaction, the enthalpy of binding (ΔH) and the association constant (K_a_), which is used to derive the binding affinity dissociation constant (K_D_).

### Transmission electronic microscopy (TEM) and immunoelectron microscopy (IEM)

Cells for TEM were processed as previously described elsewhere^36,37^. In brief, cell pellets were fixed with 2.5% glutaraldehyde in 0.1 M cacodylate buffer (pH 7.2~7.4) with gentle agitation at 4 °C overnight. Samples were washed twice in 0.1 M cacodylate buffer, and fixed again with 0.1% osmium tetroxide in 0.1 M cacodylate buffer at room temperature for 2 h. Samples were then washed twice in 0.1 M cacodylate buffer and embedded in Spurr resin (Electron Microscopy Sciences). Thin sections with a thickness of 70 nm were obtained with an ultramicrotome (Leica EM UC7). For IEM, thin sections briefly washed in PBS were blocked with 3% bovine serum albumin (BSA) and 0.2% Tween-20 in PBS at room temperature. Thin sections were reacted with the mouse anti-HA antibody (30×) at 4 °C overnight, and sequentially washed in high-salt Tween buffer (HST) (0.5 M NaCl and 0.1% Tween-20 in 50 mM Tris-HCl at pH 7.5) and PBS. Thin sections were reacted with a donkey anti-mouse IgG antibody conjugated with 12-nm gold particles (40×) (Jackson Immunoresearch) at room temperature for 1 h. Thin sections were sequentially washed with HST and PBS, fixed in 1% glutaraldehyde, washed again in PBS and distilled water, and stained with 1% osmium tetroxide and 1% uranyl acetate. Images were captured with an electron microscope (JEM 1200-EX).

Alternatively, a high-pressure freezing protocol was employed as previously described^38^, with some modifications. In brief, cell pellets washed once in PBS were re-suspended in cryoprotectant containing 1-Hexadecene and 20% BSA for 5 min at 4 °C. Samples were transferred to liquid nitrogen using a sequential cryofixation instrument (Leica EM PACT2 HPF unit) for freeze substitution, starting with 0.2% uranyl acetate, and then in acetone containing 0.2% uranyl acetate, 5% H_2_O, and 4% methanol at −85 °C for 2 days (Leica EM AFS2). Samples were warmed to −50 °C, washed with acetone, and infiltrated with Lowicryl (HM20, Electron Microscopy Sciences) at −50 °C for 1 day. Polymerization was completed by exposure to UV light at −50 °C for 2 days and at room temperature for 2 days. For IEM, thin sections washed in PBS were blocked with 5% BSA in PBS at room temperature for 20 min. Samples were double-stained with the rabbit anti-PFO (100×), anti-Myb3 (100×) or anti-*Tv*Bip (100×) antibody, along with the rat anti-*Tv*Arf-1 (100×), anti-*Tv*CyP1 (100×), or anti-*Tv*CyP2 (100×) at 4 °C overnight, and then washed in PBS containing 0.5% BSA five times at 2-min intervals. Thin sections were then reacted with the goat anti-rat IgG conjugated with 18-nm or goat anti-rabbit IgG conjugated with 12 nm gold particle (40×) (Jackson Immunoresearch) at room temperature for 1 h. Thin sections were sequentially washed in distilled water five times at 2-min intervals, and stained with 1% osmium tetroxide and 1% uranyl acetate. Images were captured with an electron microscope (FEI Tecnai G2 F20 S-TWIN).

### Subcellular fractionation by detergent

Cell lysates were fractionated into cytosolic and nuclear fractions using a subcellular fractionation kit (NE-PER™ Nuclear and Cytoplasmic Extraction Reagents, ThermoFisher Scientific).

### Subcellular fractionation by differential and gradient centrifugation

Organelle fractions were purified from 250 ml of cells for biochemical characterizations by differential and gradient centrifugation procedures as previously described^39^, with some modifications. Briefly, the postnuclear lysate was processed by differential centrifugation into crude membrane fractions, P15 and P100, and the soluble S100 fraction. The P15 fraction was layered on top of an Optiprep (Abbott Diagnostics Technologies) gradient gel generated by a step-wise (2%) increase from 18% to 36%. Samples were centrifuged at 2 × 10^5^ × *g* and 4 °C for 2 h (Beckman, SW60). Every 250-μl fraction was sampled from the gradient, referred to as P15-1, in which the top fluffy fraction was layered onto a three-layer (10%, 15%, and 25%) Optiprep gel and re-fractionated at 3×10^5^ × *g* and 4 °C for 2 h (Beckman SW60). The gradient, referred to as P15-2, was fractionated into 250-μl fractions from the top. The P100 pellet was re-suspended in 0.5 ml of buffer by sonication, mixed with 0.1 ml of 60% OptiPrep, and layered onto a OptiPrep gradient gel (12~30%), which was formed by a step-wise 2% increase in each layer. Samples were centrifuged at 3.53 × 10^5^ × *g* and 4 °C for 4 h (Beckman SW60 rotor). The sample was fractionated into 200-μl fractions starting from the top of the gradient.

### Immunoprecipitation

For the P15 and P100 samples, proteins were extracted by buffer II of the ProteoExtract® Subcellular Proteome Extraction Kit (Merck). Extracts were 5-fold diluted in PBS. Otherwise, 7.5 × 10^7^ cells were lysed in 1% TritonX-100, 1× protease inhibitor cocktail, and 200 μg ml^−1^ of TLCK in PBS. For each sample, 20 μl of the agarose-conjugated anti-HA antibody (Sigma) was added, and reactions were incubated at 4 °C overnight with constant agitation. Agarose beads recovered from low-speed centrifugation were washed three times for 10 min each in PBS containing 0.1% Triton X-100. The precipitants were recovered and boiled for 10 min for the Western blot analysis.

## Results

### Identification of *Tv*CyP2 as a Myb3-binding protein

Myb3 was previously demonstrated to interact with *Tv*CyP1 for trafficking through particular membrane compartments towards the nucleus^18^. To study other Myb3-interacting proteins, an expression library constructed in pTRG was screened using pBait-Myb3(N). Thirteen cDNA clones identical to the previously reported pTRG-c22^18^, which harbors the full-length insert of *TvCyP1*, and a unique cDNA clone, pTRG-c102, were obtained. No positive cDNA clone was obtained from screening the same library with pBait-Myb3(C), indicating that the N-terminal region of Myb3 is essential and sufficient for the detected interaction. Utilizing a pair-wise two-hybrid interaction assay, pTRG-c102 was found to interact with pBait-Myb3 and pBait-Myb3(N), but not with pBait-Myb3(C) (Fig. 1A). In contrast, pTRG-c22 was found to interact with pBait-Myb3(N).

Sequence analysis of pTRG-c102 revealed a *TvCyP2* gene (TVAG_062520), which shares 71% sequence identity with *TvCyP1* at the protein level. *TvCyP2* encodes an open reading frame of 186 amino acids (aa), with a mass estimated at ~20 kDa and a pI value of 9.18. It has a CsA-binding motif crucial for both enzymatic proficiency and CsA binding, with conserved amino acids in the catalytic pocket (Arg^75^, Phe^80^, Met^81^, Gln^83^, Gly^92^, Ala^121^, Asn^122^, Ser^123^, Gln^131^, Phe^133^, Trp^141^, Lys^142^, and His^146^), except for Ser^123^, which in other *h*CyPA homologues is Ala. Similar to *Tv*CyP1, *Tv*CyP2 also possesses an extra loop region (aa 61~67), which is conserved in *ce*CyP3 and a few other *h*CyPA homologues of worms and plants^40^ (Fig. 1B). *Tv*CyP2 has two Gly-Pro dipeptide motifs, one at ^114^Gly-Pro^115^, which is conserved in *ce*CyP3 and *h*CyPA, and another at ^124^Gly-Pro^125^, which is also present in *Tv*CyP1, *y*CPR1, and *h*CyPA. Unlike other members of the CyPA family, *Tv*CyP2 contains a unique N-terminus rich in hydrophobic amino acids (Fig. 1B).

### Enzymatic proficiency

Since His-*Tv*CyP2 was precipitated at 10 °C (data not shown), the temperature used in the enzymatic proficiency assay, GST-*Tv*CyP2 was employed for the assay. When reacting with a peptide substrate, the enzymatic proficiency (*k*_cat_/*K*_M_) of GST-*Tv*CyP2 was 4.5 μM^−1^s^−1^ (Fig. 2A), but that of GST-*Tv*CyP2(R75A) was barely detectable (Fig. 2B). In addition, the enzymatic proficiency of GST-*Tv*CyP2 was inhibited by CsA in a dose-dependent manner, with an IC_50_ of 5 nM (Fig. 2C), suggesting that *Tv*CyP2 has a typical enzymatic proficiency conferred by the conserved catalytic domain.

### *Tv*CyP2 and Myb3 interaction

**Figure 3:**
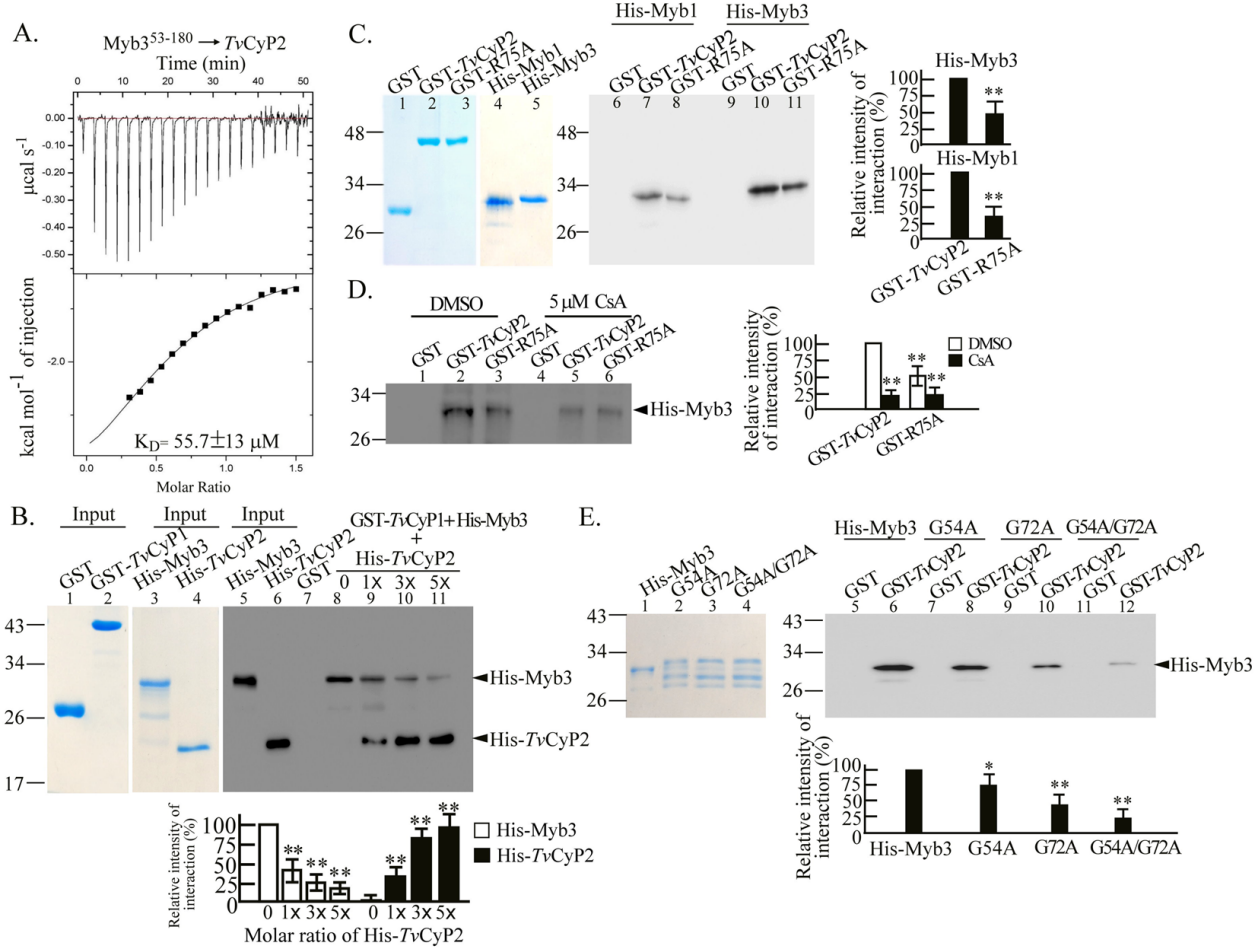
Interactions of Myb3 with *Tv*CyP2 or *Tv*CyP1. The interactions between Myb3 and *Tv*CyP2 was first examined by the ITC assay. In (**A**), a fixed amount of His-*Tv*CyP2 was titrated with increasing amounts of His-Myb3^53–180^. The K_D_ value measured was shown in the panel. For the competition assay, His-Myb3 was incubated with GST or GST-*Tv*CyP1 for 30-min, and different amounts of His-*Tv*CyP2 was added into the reaction mixtures for another 30-min. (**B**) The protein-protein interactions were then confirmed by the GST pull-down assay (**C**–**E**). In C, GST, GST-*Tv*CyP2, and GST-*Tv*CyP2(R75A) were each incubated with His-Myb3 or His-Myb1. In D, GST, GST-*Tv*CyP2, or GST-*Tv*CyP2(R75A) was incubated with His-Myb3, DMSO or 5 μM cyclosporine A (CsA). In E, GST and GST-*Tv*CyP2 were each incubated with His-Myb3, Myb3(G54A), Myb3(G72A), or Myb3(G54A/G72A). Protein samples were separated by SDS-PAGE for Coomassie blue staining (the left panel of B, C, and E). In a duplicate gel, 1/10 of the input proteins or 1/5 of the pulled down products were examined by the Western blotting using the anti-6 × His antibody (D, and the right panel of B, C, and E). Relative signal intensities of Western blotting were quantified as shown in the histograms. *p < 0.05, **p < 0.01. Error bars represent the standard deviation (*n* = 3).

ITC was then employed to study the protein-protein interaction of *Tv*CyP2 or *Tv*CyP1 with Myb3. Since the full-length His-Myb3 was easily degraded during purification, His-Myb3^53–180^ previously used for the structural analysis of Myb3^24,35^, was employed in the assay. When a fixed amount of His-*Tv*CyP2 was titrated with increasing amounts of His-Myb3^53–180^, exothermic heat changes were measured with a binding affinity dissociation constant (K_D_) calculated to be ~55 μM (Fig. 3A). By contrast, protein aggregation was observed when a fixed level of His-*Tv*CyP1 was titrated with increasing amounts of His-Myb3^53–180^. (Supplementary Fig. 19A). The interaction between His-*Tv*CyP1 and His-*Tv*CyP2 is too weak to be measured by ITC (Supplementary Fig. 19B).

Thus, the competition of *Tv*CyP1 and *Tv*CyP2 against Myb3 was further studied by the GST-pull down assay (Fig. 3B). In this assay, preformed protein complex comprising *Tv*CyP1 and Myb3 was titrated with *Tv*CyP2. Decreasing amounts of His-Myb3 were detected with increasing input levels of His-*Tv*CyP2, suggesting that *Tv*CyP2 can compete with *Tv*CyP1 for the binding to Myb3 under our test conditions.

The protein-protein interactions were confirmed by a GST pull-down assay. To do this, GST, GST-*Tv*CyP2, and GST-*Tv*CyP2(R75A) were each reacted with His-Myb3 or His-Myb1. Pull-down products were examined by the Western blotting using the anti-6xHis antibody. A ~32-kDa His-Myb3 band was detected in the products pulled down by GST-*Tv*CyP2 and GST-*Tv*CyP2(R75A), but not GST (Fig. 3C), with a slightly higher level of Myb3 detected in samples pulled down by GST-*Tv*CyP2 than by GST-*Tv*CyP2(R75A). However, the binding of His-Myb3 to either GST-*Tv*CyP2 or GST-*Tv*CyP2(R75A) was greatly diminished in the presence of 5 μM CsA (Fig. 3D), implying that the catalytic domain of *Tv*CyP2 is essential for binding to Myb3, but additional elements may be required for optimal binding of *Tv*CyP2 to Myb3.

The GP dipeptide motifs in Myb1 and Myb3 are crucial for binding to *Tv*CyP1^18^. We speculated that ^54^GP^55^ and ^72^GP^73^ in Myb3 might also be crucial for binding to *Tv*CyP2. To test this possibility, mutant proteins were included in the GST pull-down assay. GST-*Tv*CyP2, but not GST, was found to bind to His-Myb3 at a level slightly higher than that with His-Myb3(G54 A) or His-Myb3(G72A), but the binding of GST-*Tv*CyP2 to His-Myb3(GG54/72AA) was aborted (Fig. 3E). These results suggest that Myb3 may have at least two *Tv*CyP2-binding motifs, each of which spans a GP dipeptide, but either one of them is sufficient to interact with *Tv*CyP2. Similar results were observed for binding of *Tv*CyP1 to His-Myb3^24^. By contrast, no signal was detected in the pull down products when individual His-tagged proteins were separately or simultaneously reacted with the glutathione-conjugated beads (Supplementary Fig. 20). Together, these observations suggest that *Tv*CyP1 and *Tv*CyP2 may compete for the same binding sites in Myb3.

### Subcellular localization of *Tv*CyP2

**Figure 4:**
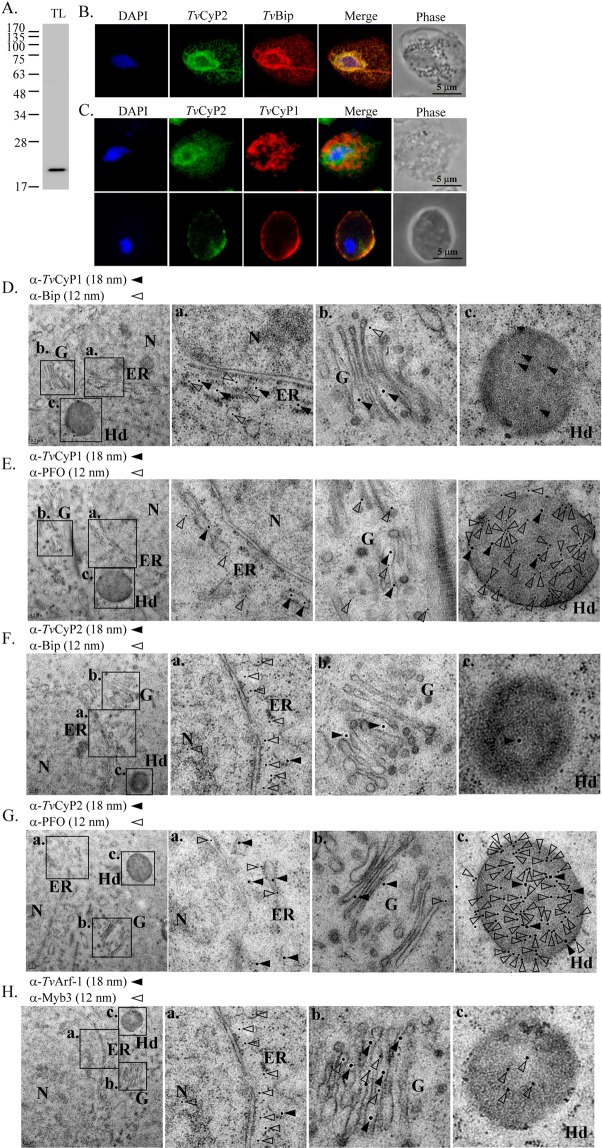
Subcellular localization of *Tv*CyP2 in *Trichomonas vaginalis*. In (**A)**, cell lysates from *T. vaginalis* in normal growth medium were examined by Western blotting using the anti-*Tv*CyP2 antibody. In (**B,C**), an IFA of *T. vaginalis* cells was performed using the rat anti-*Tv*CyP2, rabbit anti-*Tv*Bip, and mouse anti-*Tv*CyP1 antibodies as indicated. Cells with (B and C top panel) or without (C, bottom panel) detergent permeation were then reacted with fluorescence-conjugated secondary antibodies. Nuclei were stained with DAPI. Fluorescence signals were recorded under confocal microscopy and merged. Cell morphology was recorded by phase-contrast microscopy. Bars in the micrographs represent 5 μm. In **D**–**H**), subcellular localization of *Tv*CyP2, *Tv*CyP1 or Myb3 was examined by the immunoelectron microscopy. Thin sections were double-stained by the rat anti-*Tv*CyP1 and rabbit anti-*Tv*Bip (**D**), rat anti-*Tv*CyP1 and rabbit anti-PFO (**E**), rat anti-*Tv*CyP2 and rabbit anti-*Tv*Bip (**F**), rat anti-*Tv*CyP2 and rabbit anti-rabbit PFO (**G**), or rat anti-*Tv*Arf-1 and rabbit anti-PFO (**H**). After washing, thin sections were reacted with the anti-rat IgG conjugated with 18-mm gold particles (closed triangles) and the anti-rabbit IgG conjugated with 12-nm gold particles (opened triangles). N, nucleus; ER, endoplasmic reticulum; (**G**), Golgi complex; H, hydrogenosome.

The specificity of the anti-*Tv*CyP2 antibody against *Tv*CyP2 was assayed by Western blotting (Fig. 4A). A single ~20-kDa band was identified in the blot from cell lysates, indicating that the antibody is specific to *Tv*CyP2. The subcellular localization of *Tv*CyP2 was then studied by an IFA. When paraformaldehyde-fixed cells permeated with detergent were double-stained with the anti-*Tv*CyP2 and anti-*Tv*Bip antibodies (Fig. 4B), *Tv*CyP2 and *Tv*Bip, a protein marker for the ER (P. Johnson, personal communication), were localized to the ER, but with much weaker signals extending extensively into the cytoplasm. When signals from each antibody were superimposed, overlapping images were observed, indicating that *Tv*CyP2 is primarily an ER protein. When fixed cells were permeated with detergent and double-stained with the anti-*Tv*CyP2 and anti-*Tv*CyP1 antibodies (Fig. 4C), *Tv*CyP2 was localized to the ER and *Tv*CyP1 to hydrogenosomes. When fixed cells without prior permeation were double-stained with the same antibodies, the *Tv*CyP2 and *Tv*CyP1 signals partially overlapped on the plasma membrane.

Subcellular co-localization of *Tv*CyP1, *Tv*CyP2 or Myb3 with selected organelle markers was further investigated by the immunoelectron microscopy (Fig. 4D,H). In these experiments, *Tv*Bip, *Tv*Arf1 and PFO were taken as the markers for the ER, Golgi and hydrogenosomes, respectively, albeit multiple subcellular localizations for each of the organelle markers. In thin sections double-stained by the anti-*Tv*CyP1 and anti-*Tv*Bip antibodies (Fig. 4D), *Tv*CyP1 and *Tv*Bip were mostly localized to the ER, and to a much less extent also detected in the Golgi. A substantial amount of *Tv*CyP1, but not *Tv*Bip, was also localized to the hydrogenosomes. In thin sections double-stained with the anti-*Tv*CyP1 and anti-PFO antibodies (Fig. 4E), *Tv*CyP1 and PFO were mostly co-localized to the hydrogenosomes, but they were also detected in the ER and Golgi to much lesser extents. *Tv*CyP2 and *Tv*Bip were also co-localized to the ER in thin sections double-stained by the anti-*Tv*CyP2 and anti-*Tv*Bip antibodies, but only the former was detected in the Golgi and hydrogenosomes (Fig. 4F). *Tv*CyP2 and PFO were also co-localized to the ER, Golgi and hydrogenosomes (Fig. 4G). By contrast, co-localization of Myb3 and *Tv*Arf-1 was detected in the ER and Golgi in thin section double-stained by the anti-*Tv*Arf-1 and anti-Myb3 antibodies, whereas Myb3, but not *Tv*Arf-1, was also detected in the hydrogenosomes, cytoplasm and nucleus (Fig. 4H). Given that *Tv*CyP1, *Tv*CyP2, *Tv*Bip and *Tv*Arf1 were rarely detected in the cytoplasm and nucleus, our observations suggest *Tv*CyP1, *Tv*CyP2 and Myb3 probably co-exist in the ER, Golgi and hydrogenosomes for interactions in a spatial and temporal manner to fulfill their functional roles in *T. vaginalis*.

### Subcellular distribution of *Tv*CyP2

**Figure 5:**
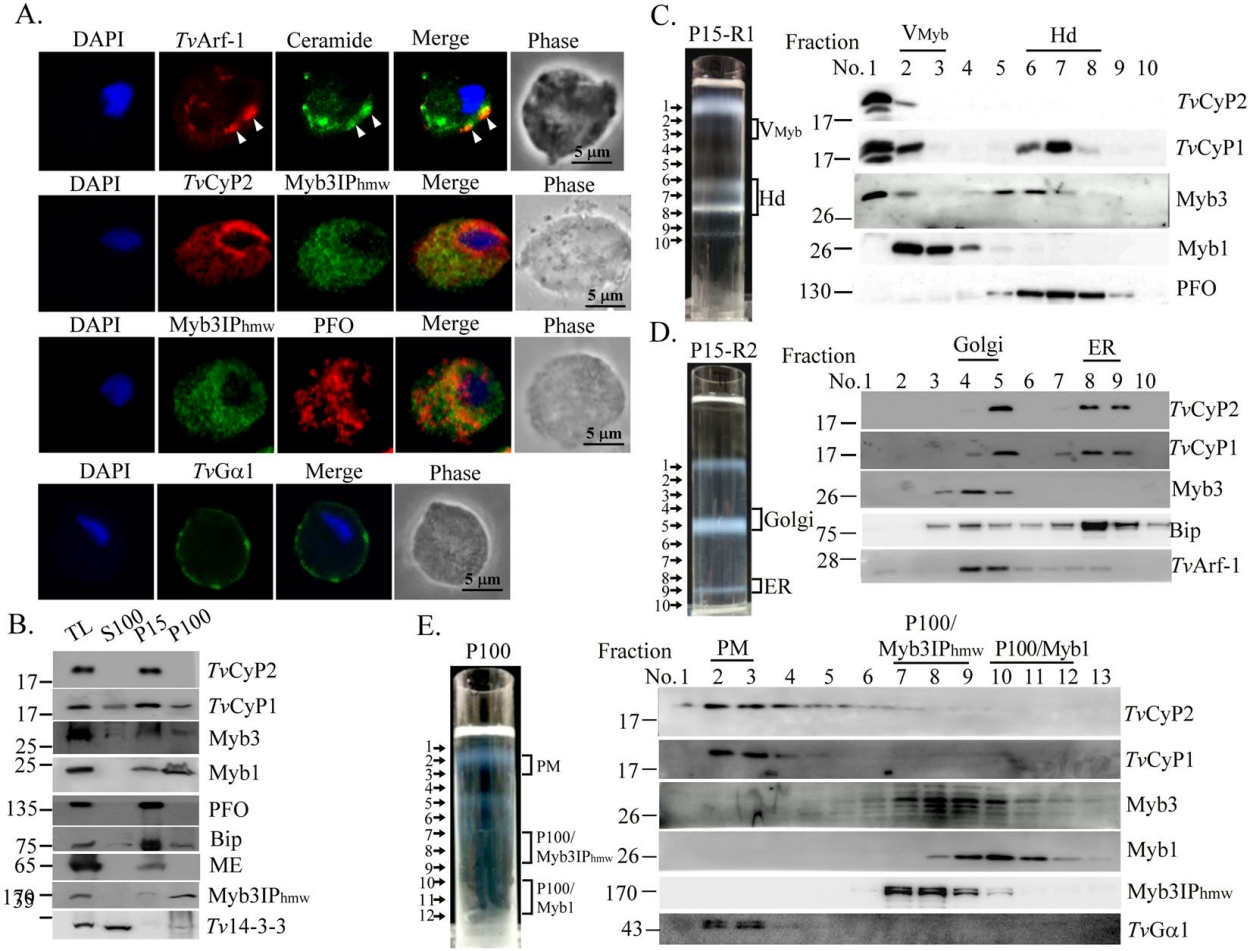
Subcellular distribution of *Tv*CyP2. In (**A**), subcellular localizations of various organelle markers were determined by an IFA. To do this, fixed cells were double-stained with the anti-*Tv*Arf-1 and ceramide (the top panels), anti-*Tv*CyP2 and Myb3IPhmw (panels in the second row), anti-Myb3IPhmw and anti-PFO (panels in the third row), or anti-*Tv*Gα1 (bottom panels) antibodies. Nuclei were stained with DAPI. Fluorescent signals were recorded by confocal microscopy. In B, total lysates (TL) from T1 were separated into soluble (S100) and crude membrane fractions, P15 and P100, by differential centrifugation. In this blot, *Tv*14-3-3, malic enzyme (ME), and Myb3IPhmw were used as respective markers for the S100, P15, and P100 fractions. In (**C,D**), P15 was fractionated by the first gradient centrifugation, with thick fluffy materials on top (C, left panel). The fluffy materials were fractionated by a second gradient centrifugation (D, left panel). In (**E**), P100 was fractionated by gradient centrifugation (left panel). A 250- (**C,D**) or 200-μl (**E**) aliquot was collected from the top of each gradient for Western blotting using antibodies indicated on the right hand side (**B,C**–**E** right panels).

Myb3IP_hmw_, Myb1, and some hydrogenosomal proteins were used as protein markers for the membrane fractionation experiments^18,24^. To identify other organelle markers for membrane fractions not defined earlier^24^, subcellular localization of *Tv*Arf-1, Myb3IP_hmw_, and *Tv*Gα1 was studied by an IFA (Fig. 5A). When fixed cells were reacted with the anti-*Tv*Arf-1 antibody and ceramide, a fluorescent marker of the Golgi complex^41^, overlapping signals were observed in structures adjacent to the nucleus, indicating that *Tv*Arf-1 may be used as a marker for the Golgi complex. When the IFA was performed using the anti-*Tv*Gα1 antibody, the signal was localized on the plasma membrane, indicating that *Tv*Gα1 can be used as a marker for plasma membrane fractions. When fixed cells were co-stained with the anti-*Tv*CyP2 and anti-Myb3IP_hmw_ antibodies, *Tv*CyP2 was localized in the ER and extended extensively as a tubular network-like structure into the cytoplasm, and Myb3IP_hmw_ was localized to a similar cytoplasmic network. The co-localization of *Tv*CyP2 and Myb3IP_hmw_ was observed at certain spots in this network. When fixed cells were double-stained with the anti-Myb3IP_hmw_ and anti-PFO antibodies (Fig. 5A), Myb3IP_hmw_ was localized adjacent to PFO. These observations suggest that the Myb3IP_hmw_ network may extend from the ER into regions in close proximity to hydrogenosomes and other organelles.

To study the membrane distribution of *Tv*CyP2, lysates were separated into the membrane fractions, P15 and P100, and the soluble S100 fraction, by differential centrifugation for Western blotting (Fig. 5B, Supplementary Fig. 21A). *Tv*CyP2 was enriched in the P15 fraction, but not in S100 or P100. When the P15 fraction was further separated by gradient centrifugation, *Tv*CyP2 was mostly detected in the fluffy materials at the top of the gradient (Fig. 5C), with a slight amount in fraction 2, the V_myb_ fraction^24^. The fluffy fraction was separated by a second gradient centrifugation into three distinct bands in fractions 1, 5, and 9 (Fig. 5D). As determined by the Bradford assay, proteins were mostly enriched in fractions 5~9 (data not shown). When samples were examined by Western blotting, *Tv*CyP2 was enriched in fractions 5, 8, and 9. *Tv*CyP1 was enriched in fraction 5, but at much lower levels also in fractions 8 and 9. In contrast, *Tv*Bip was detected in fractions 4~10, but mostly in fraction 8. Samples taken from fractions 1~10 were examined by LC-MS/MS-based proteomics. Six unique peptides of a particular calreticulin (TVAG_120870), an ER marker in eukaryotic model organisms^42^, were detected only in samples from fraction 9 (C. H. Chu and J. H. Tai, unpublished data), suggesting that this fraction is truly enriched in the ER. Since *Tv*Arf-1 was localized to the Golgi complex (Fig. 5D), fractions 4 and 5 are reputed to be the Golgi fraction in this text. Since Myb3 was detected in fractions 4 and 5, it may reside in the Golgi complex before being sorted into a particular membrane trafficking pathway. When the P100 fraction was fractionated by gradient centrifugation (Fig. 5E), *Tv*CyP2 was detected in fractions 2~6, while *Tv*CyP1 and *Tv*Gα1 were mainly detected in fractions 2 and 3. When pooled together, these fractions are reputed to be plasma membrane fractions. Consistent with our previous findings, Myb3 and Myb1 were respectively enriched in the P100/Myb3IP_hmw_ and P100/Myb1 fractions^24^.

### *Tv*CyP2 and the nuclear translocation of Myb3

**Figure 6:**
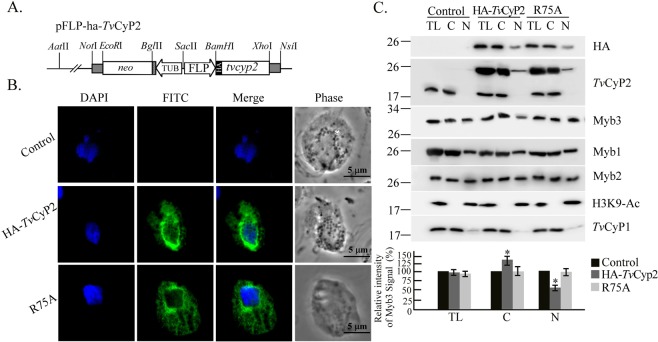
*Tv*CyP2 and nuclear translocation of Myb3. The stably expressing plasmid, pFLP-ha-*Tv*CyP2, that overexpresses HA-*Tv*CyP2 in *Trichomonas vaginalis* is depicted in (**A**). A mutation was introduced to produce pFLP-ha-*Tv*CyP2(R75A). In (**B**), transfected cells overexpressing HA-*Tv*CyP2, HA-*Tv*CyP1(R75A), and non-transfected controls were sequentially reacted with the rat anti-HA antibody paired with FITC-conjugated rat IgG. Nuclei were stained with DAPI. Fluorescence signals (FITC and DAPI) were recorded under confocal microscopy and merged. Cell morphology was recorded under phase-contrast microscopy. Bars in the micrographs represent 5 μm. In **C**, cell lysates (TL) from cells overexpressing HA-*Tv*CyP2 or HA-*Tv*CyP2(R75A), and control cells were fractionated into cytosolic (**C**) and nuclear (N) fractions for Western blotting using antibodies for detecting various proteins as indicated. The relative intensities of Myb3 versus H3K9-Ac or *Tv*CyP1 in the nuclear or cytosolic samples are quantified as shown in the histograms at the bottom. *p < 0.05. Error bars represent the standard deviation (*n* = 3).

To study the role of *Tv*CyP2 in the nuclear translocation of Myb3, plasmids overexpressing HA-*Tv*CyP2 and the enzymatic activity-deficient mutant, *Tv*CyP2(R75A), were constructed (Fig. 6A). Cells overexpressing HA-*Tv*CyP2 and *Tv*CyP2(R75A) were established. When examined by an IFA (Fig. 6B), the subcellular localization of HA-*Tv*CyP2 and *Tv*CyP2(R75A) stained with the anti-HA antibody was similar to that when stained with the anti-*Tv*CyP2 antibody. To test whether *Tv*CyP2 regulates the nuclear translocation of Myb3, cells were fractionated by a detergent-based protocol into cytosolic and nuclear fractions for Western blotting (Fig. 6C). The purities of the cytosolic and nuclear fractions were validated by detecting *Tv*CyP1 and H3K9-Ac, respectively, in these fractions. HA-*Tv*CyP2 and *Tv*CyP2(R75A) were each detected at similar levels in the cytosolic and nuclear fractions only from transgenic cells. Given that *Tv*CyP1 was only detected in samples from total lysates and cytosolic fractions, a slight amount of *Tv*CyP2 in the nuclear fractions might not have been due to cross-contamination during sample preparation. HA-*Tv*CyP2 or *Tv*CyP2(R75A) was overexpressed at a level 3~5-fold higher than endogenous one. Expression of HA-*Tv*CyP2 or *Tv*CyP2(R75A) had slight effects on the overall expressions of other proteins examined in the blot. The amount of nuclear Myb3 was much lower in samples from cells overexpressing HA-*Tv*CyP2 than from control cells, and the decrease was partially reverted in samples from *Tv*CyP2(R75A)-overexpressing cells. The amounts of Myb1 and Myb2 in the cytosolic and nuclear fractions only slightly varied in these samples, suggesting that *Tv*CyP2 may downregulate the nuclear translocation of Myb3 in a manner depending on its enzymatic proficiency.

### *Tv*CyP2 and protein membrane trafficking

**Figure 7:**
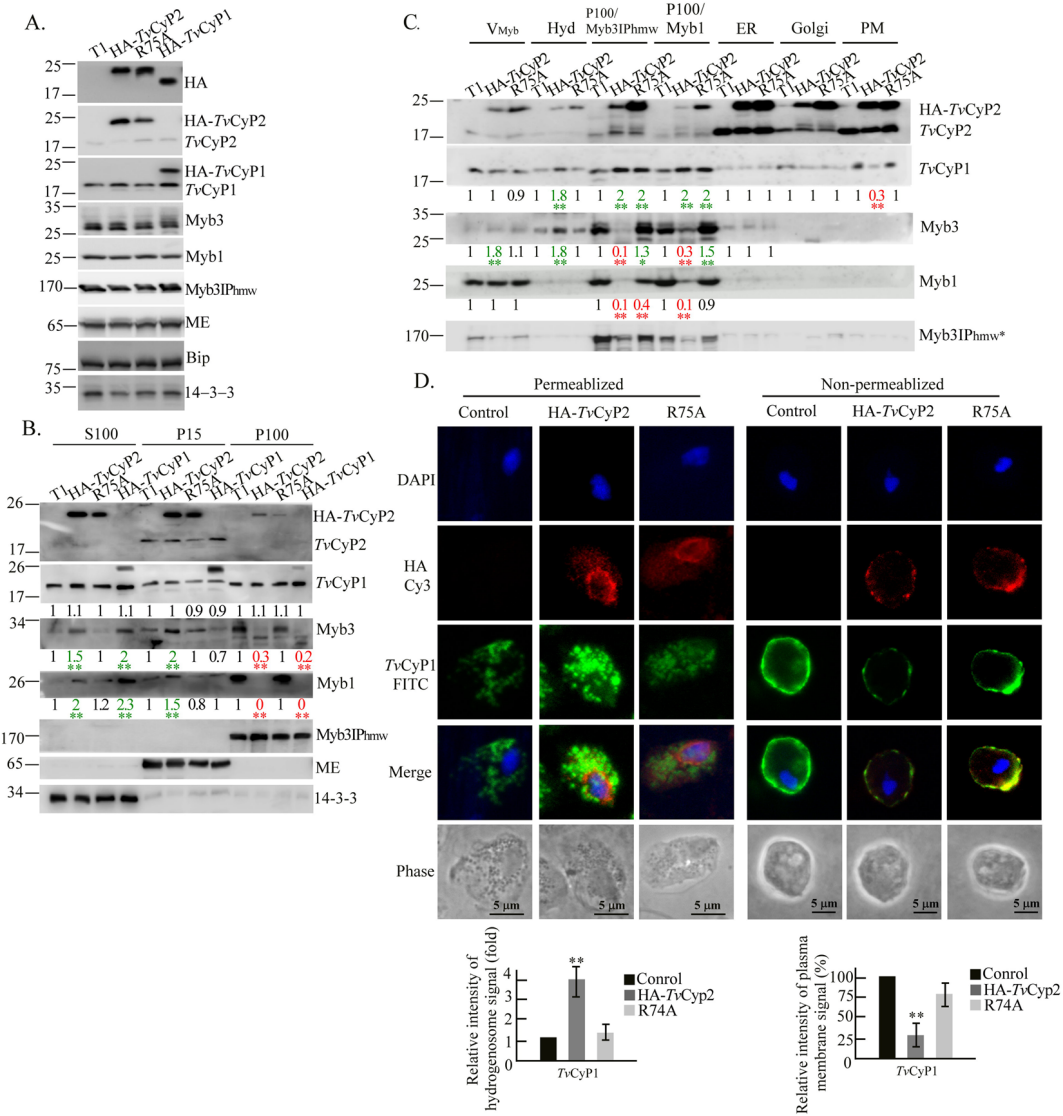
*Tv*CyP2 and the subcellular distribution of membrane-bound Myb3. Samples from total lysates (**A**), crude cellular fractions (**B**), and enriched organelle fractions (**C**) from control cells (T1) and cells overexpressing HA-*Tv*CyP2, HA-*Tv*CyP2(R75A), or HA-*Tv*CyP1 as indicated on top of each panel were examined by Western blotting using various antibodies to detect selected proteins as indicated on the right side of each panel. Molecular weights are indicated on the left side of each panel. Relative intensities of the protein bands in Western blotting from three independent experiments are quantified as shown at the bottom of each panel. *p < 0.05, **p < 0.01. In (**D**), cells with or without detergent permeation were double-stained with anti-HA and anti-*Tv*CyP1 antibodies followed by secondary antibodies conjugated with FITC and Cy3. Nuclei were stained with DAPI. Signal intensities of *Tv*CyP1 in hydrogenosomes and on the plasma membrane are quantified as shown in the histograms at the bottom. *p < 0.05, **p < 0.01. Error bars represent the standard deviation (*n* = 3).

Since *Tv*CyP2 was only detected in membrane fractions, it may regulate the transition of Myb3 through membrane compartments. To address this question, total lysates were first examined by Western blotting (Fig. 7A). Overexpression of HA-*Tv*CyP2, *Tv*CyP2(R75A), and HA-*Tv*CyP1 exerted slight effects on the overall protein expression.

Lysates were then separated into the P15, P100, and S100 fractions for Western blotting (Fig. 7B, Supplementary Fig. 21B). HA-*Tv*CyP2 and *Tv*CyP2(R75A) were each detected at similar levels in the S100 and P15 fractions, but only at much lower levels in the P100 fraction. Significant amounts of HA-*Tv*CyP2 and *Tv*CyP2(R75A) detected in the S100 fraction may have been due to their excessive overexpression. HA-*Tv*CyP1 was mostly enriched in the P15 fraction, but to a lesser extent in the S100 fraction, with only a trace amount in the P100 fraction. Its level in various fractions changed slightly in samples from different cell lines. Overexpression of HA-*Tv*CyP2 resulted in an increasing level of Myb3 or Myb1 in the S100 and P15 fractions, with a concurrent lower level in the P100 fraction, but these effects were not seen in samples from cells overexpressing *Tv*CyP2(R75A). Together, these observations suggest that *Tv*CyP2 may accelerate the transition of Myb1 and Myb3 through various membrane compartments that rely on its enzymatic proficiency.

The P15 and P100 fractions were further fractionated by gradient centrifugation, and the same organelle fractions were pooled for Western blotting (Fig. 7C). HA-*Tv*CyP2 and *Tv*CyP2(R75A) were each detected at similar levels in the ER and plasma membrane fractions, but higher levels of *Tv*CyP2(R75A) were detected in membrane fractions enriched with the Golgi complex, P100/Myb3IP_hmw_, P100/Myb1, and V_myb_, indicating that the enzymatic proficiency of *Tv*CyP2 is crucial for its own membrane trafficking. It was notable that slight amounts of HA-*Tv*CyP2 and *Tv*CyP2(R75A) were consistently detected in hydrogenosomal fractions. This might not have been due to cross-contamination during sample preparation, since *Tv*CyP2 was localized to hydrogenosomes by IEM (Fig. 4G). Myb3 was detected at levels higher in the V_myb_ and/or hydrogenosomal fractions, but was lower in the P100/Myb3IP_hmw_ and P100/Myb1 fractions, in samples from cells overexpressing HA-*Tv*CyP2 compared to cells overexpressing *Tv*CyP2(R75A). Similar effects of transfected proteins on the distribution of Myb1 were observed in the P100/Myb3IP_hmw_ and P100/Myb1 fractions, but not in the V_myb_ fraction. Moreover, Myb3IP_hmw_ was detected in the V_myb_, P100/Myb3IP_hmw_, and P100/Myb1 fractions at levels lower in samples from cells overexpressing HA-*Tv*CyP2 compared to cells overexpressing *Tv*CyP2(R75A) or control cells, indicating that Myb3IP_hmw_ may also undergo membrane trafficking regulated by *Tv*CyP2. A higher than control level of *Tv*CyP1 was detected in the P100/Myb3IP_hmw_ and P100/Myb1 fractions in samples from cells overexpressing HA-*Tv*CyP2 or *Tv*CyP2(R75A), indicating that the membrane trafficking of *Tv*CyP1 towards the P100/Myb3IP_hmw_ and P100/Myb1 membrane compartments might not fully depend on the enzymatic proficiency of *Tv*CyP2.

An IFA was used to confirm results from the fractionation experiments. To do this, fixed cells were double-stained with the anti-HA and anti-*Tv*CyP1 antibodies (Fig. 7D). After detergent permeation, *Tv*CyP1 was detected in the hydrogenosomes at a level higher in cells overexpressing HA-*Tv*CyP2 than in control cells. Without detergent permeation, *Tv*CyP1 was detected on the plasma membrane at a level lower in cells overexpressing HA-*Tv*CyP2 than in control cells. In these experiments, the effects of HA-*Tv*CyP2 were partially reversed by *Tv*CyP2(R75A), suggesting that *Tv*CyP2 may regulate the trafficking of some proteins among distinct membrane compartments, like Myb3 and *Tv*CyP1 to hydrogenosomes, Myb3IP_hmw_ to the V_myb_, and the export of *Tv*CyP1 onto the plasma membrane.

### Differential complex formation

**Figure 8:**
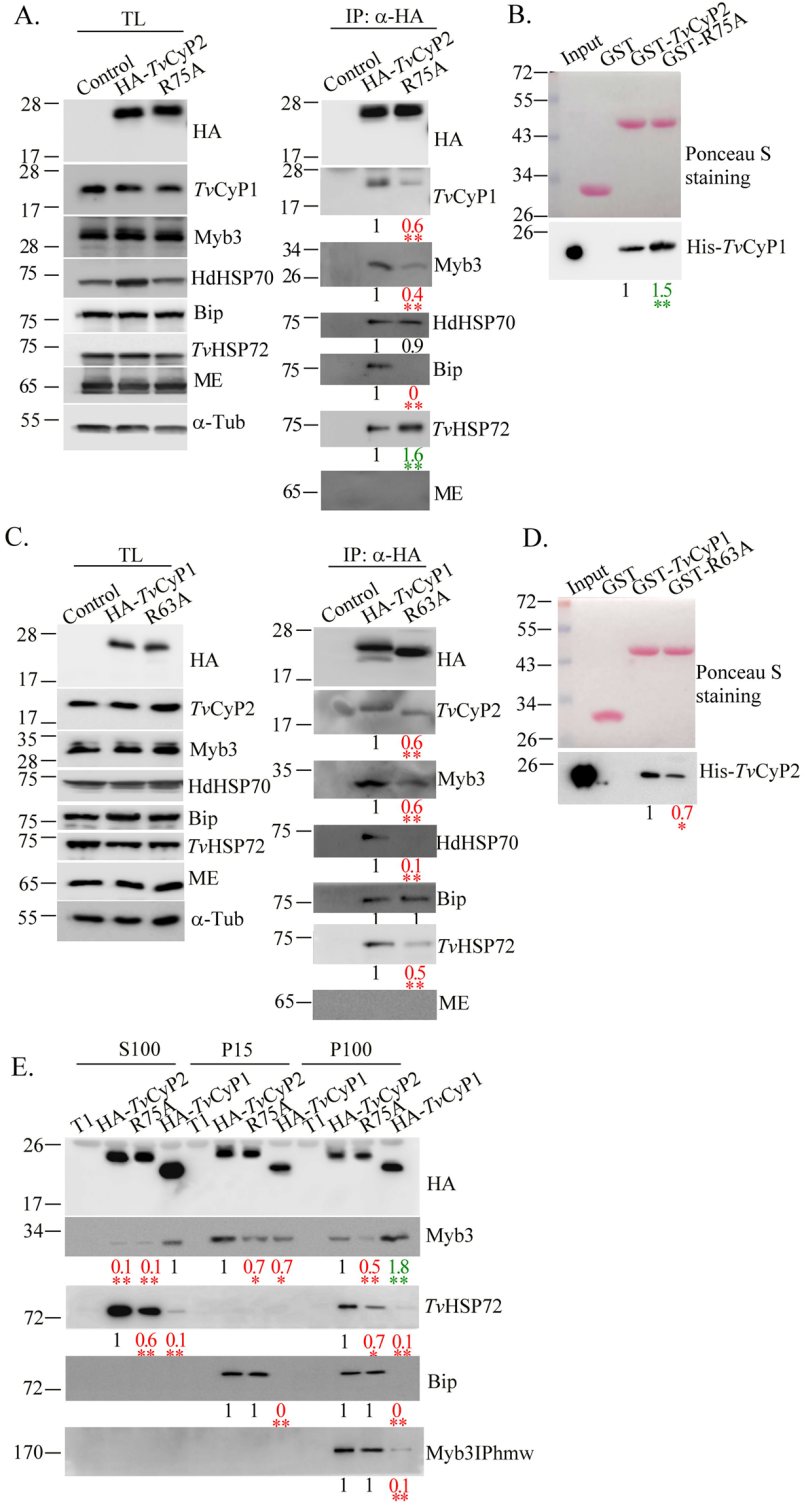
Protein complexes and protein-protein interaction between *Tv*CyP2 and *Tv*CyP1. Total lysates (**A**,C) or the P15, P100, and S100 fractions (**E**) were immunoprecipitated by an anti-HA antibody. Protein samples were examined by Western blotting to detect proteins indicated on the right side of each panel. Molecular weights are indicated on the left side of each panel. In (**B**), glutathione-S-transferase (GST), GST-*Tv*CyP2, and GST-*Tv*CyP2(R75A) were each reacted with His-*Tv*CyP1. In (**D**), GST, GST-*Tv*CyP1, and GST-*Tv*CyP1(R63A) were each reacted with His-*Tv*CyP2. Protein samples were separated by SDS-PAGE in a 12% gel for Ponceau S staining (B and D, top panels). In a duplicate gel, 1/10 of the input proteins or 1/5 of the pulled down products were examined by the Western blotting using the anti-6 × His antibody (B and D bottom panels). Relative signal intensities from three experiments are quantified as shown at the bottom of each lane. *p < 0.05, **p < 0.01.

Since *Tv*CyP2 and *Tv*CyP1 may target the same biding motifs in Myb3^18,24^, and *Tv*CyP2 contains two Gly-Pro motifs, one of which is also conserved in *Tv*CyP1 (Fig. 1B), these two cyclophilins may simultaneously interact with each other and with their common substrates. To examine this possibility, the protein complex in cell lysates was pulled down by immunoprecipitation using the anti-HA antibody. Samples were collected for Western blotting. As shown below, overexpression of HA-*Tv*CyP2 had slight effects on expressions of Myb3, *Tv*CyP1, *Tv*Bip, *Tv*HSP72, and the hydrogenosomal malic enzyme, AP65, but it possibly enhanced expression of hydrogenosomal HSP70 in a manner correlated with the enzymatic proficiency of *Tv*CyP2 (Fig. 8A, left panel). In the HA-*Tv*CyP2-complex, *Tv*CyP1, Myb3, *Tv*Bip, and *Tv*HSP72, but not hydrogenosomal HSP70, were detected at levels related to the enzymatic proficiency of *Tv*CyP2 (Fig. 8A right panel). The GST pull-down assay was used to explore whether *Tv*CyP2 directly interacts with *Tv*CyP1 (Fig. 8B). In this assay, GST and GST-fusion proteins were separately reacted with His-*Tv*CyP1, and the pull-down products were examined by Western blotting using the anti-His antibody. A duplicate gel was stained with Ponceau S to show input levels of individual proteins. A ~19-kDa band of His-*Tv*CyP1 was pulled down at a lower level by GST-*Tv*CyP2 than by GST-*Tv*CyP2(R75A), but not by GST, indicating that *Tv*CyP2 directly interacts with *Tv*CyP1 in a manner inversely correlated with the enzymatic proficiency of *Tv*CyP2.

Overexpression of HA-*Tv*CyP1 had slight effects on overall expressions of Myb3, *Tv*CyP2, *Tv*Bip, *Tv*HSP72, and hydrogenosomal HSP70 (Fig. 8C, left panel). These proteins, except for *Tv*Bip, were also detected in the HA-*Tv*CyP1 complex at levels that were correlated with the enzymatic proficiency of *Tv*CyP1 (Fig. 8C right panel). In the GST pull-down assay (Fig. 8D), His-*Tv*CyP2 was pulled down at a level significantly higher by GST-*Tv*CyP1 than by GST-*Tv*CyP1(R63A), but not by GST, indicating that *Tv*CyP1 directly interacts with *Tv*CyP2 in a manner correlated with the enzymatic proficiency of *Tv*CyP1.

To further test whether *Tv*CyP2 formed a distinct protein complex comprising Myb3 and its interacting proteins in various membrane compartments, proteins in the P15, P100, and S100 fractions from various cell lines were immunoprecipitated with the anti-HA antibody for Western blotting. As shown in Fig. 8E, Myb3 was detected in the protein complex comprising *Tv*CyP2 at a level higher in samples from the P15 fraction than the P100 fraction, but not in the S100 fraction, in a manner that correlated with the enzyme proficiency of *Tv*CyP2. *Tv*HSP72, *Tv*Bip, and Myb3IP_hmw_ were all detected in the HA-*Tv*CyP2-complex in samples from the P100 fraction, whereas *Tv*HSP72 was also detected in the S100 and P15 fractions at levels less dependent on the enzyme proficiency of *Tv*CyP2. Since the presence of *Tv*CyP2 in soluble cytosol is negligible (Fig. 5B), these results suggest that *Tv*CyP2 may regulate the transition of Myb3 among distinct compartments through the endomembrane trafficking pathway, possibly in coordination with *Tv*Bip, Myb3IP_hmw_, or *Tv*HSP72 at various sites. On the other hand, Myb3 was predominantly associated with the protein complex comprising HA-*Tv*CyP1, mostly in samples from the P100 fraction, and at a much lower level in those from the P15 and S100 fractions. The amounts of *Tv*HSP72, *Tv*Bip, and Myb3IP_hmw_ in *Tv*CyP1-complexes were barely detectable under our test conditions, suggesting that HA-*Tv*CyP1 may regulate the release of Myb3 and the Myb3-binding proteins, *Tv*HSP72 and Myb3IP_hmw_, from the protein complex.

### The fusion of vesicles with and budding of vesicles from hydrogenosomes

**Figure 9:**
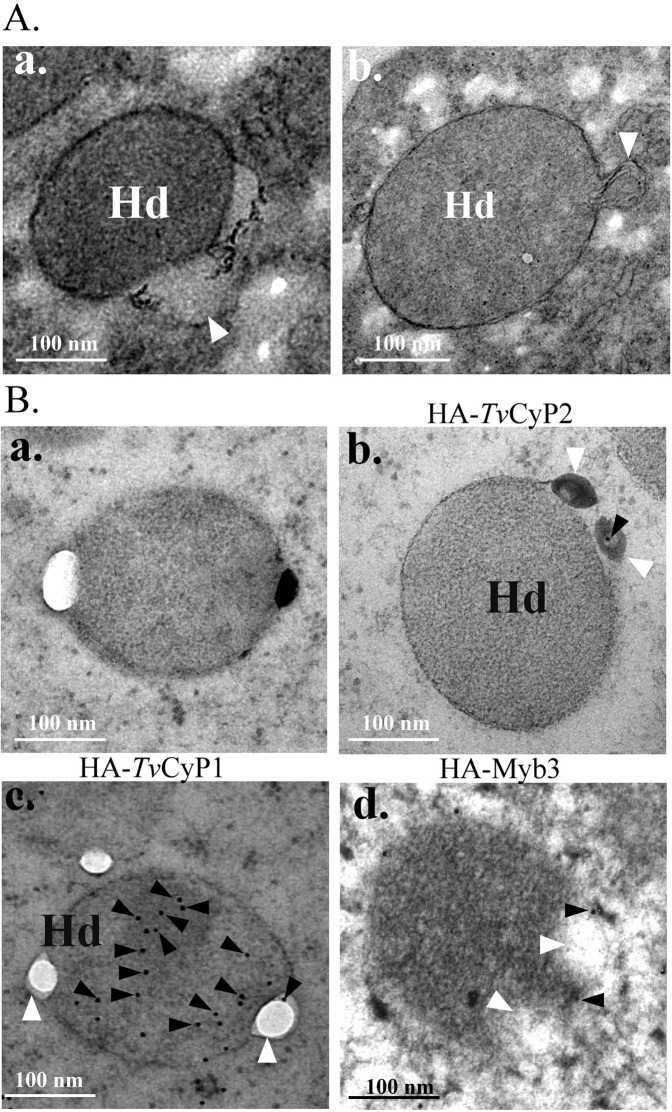
Observations of vesicles fused with or budding from hydrogenosomes. Vesicles (V) fused with (**A**-a) or budding from (**A**-b) a hydrogenosome (Hd) were observed by TEM. In B, cells after cryofixation (**B**-a, **B**-b, B-c) or chemical fixation (**B**-d) were embedded in Spurr resin. Thin sections from control cells (**B**-a) or transgenic cells overexpressing HA-*Tv*CyP2 (**B**-b), HA-*Tv*CyP1 (**B**-c), or HA-Myb3 (**B**-d) were sequentially reacted with an anti-HA antibody and 12-nm gold particles (closed triangles) conjugated to IgG. The fusion of a vesicle with or budding of a vesicle from a hydrogenosome is indicated by an open triangle. Bars in the micrographs represent 100 nm.

When examined by TEM, vesicles fused with the more-electron-dense counterpart of hydrogenosomes were rarely observed in samples from cells cultured in normal growth medium (Fig. 9A), but > 60% of hydrogenosomes fused with vesicles was consistently identified in samples from cells replete with 250 μM of iron overnight. To explore iron’s effect, thin sections from various cell lines were examined by IEM using the anti-HA antibody (Fig. 9B). Gold particles were detected at fusion junctions and in hydrogenosomes in samples from cells overexpressing HA-*Tv*CyP1, HA-*Tv*CyP2, and HA-Myb3, but not in control cells. These observations provide morphological evidence to support the notion that trafficking of some hydrogenosomal proteins occurs through the endomembrane system.

Together, our observations suggest that *Tv*CyP2 probably regulates trafficking of some proteins through the endomembrane system to the plasma membrane, the hydrogenosomes, or the nucleus.

## Discussion

Protein trafficking in a eukaryotic cell from the site of translation towards the site of function is dynamically regulated in a spatiotemporal manner^43^. For example, transcription factors with distinct nuclear localization signals are often translated onto cytosolic ribosomes to be imported into the nucleus via the Ran-mediated import machinery^44^. In *T. vaginalis*, Myb2, Myb3, and perhaps also Myb1 may exploit conserved helical structures as a dual functional entity for nuclear import and DNA binding^22,23^. Although a signaling pathway for the nuclear import of Myb3 from the cytosol upon sudden iron overload is well studied^19^, the cellular machinery for protein nuclear translocation in this parasite remains elusive. Intriguingly, Myb1 and Myb3 are mostly restricted to cytoplasmic membrane compartments^18,24^, indicating that their nuclear translocation may require a few transitional stages from the endomembrane system to reach the cytosol. In a continuing effort to understand the trafficking of membrane-bound Myb proteins^18,24^, an earlier developed protocol was herein refined to obtain purified membrane fractions enriched with some intracellular organelles, like the ER and Golgi complex, and possibly also some putative tubulovesicular transport intermediates, like V_myb_ and the P100/Myb3IP_hmw_ compartment (Fig. 5). Although the purity of each membrane fraction remains to be determined, the protocol described herein may provide a starting point to search for more protein markers to study the endomembrane system in this parasite.

Although *Tv*CyP1 and *Tv*CyP2 may have similar properties in terms of enzymatic proficiency, substrate recognition, and membrane distribution^18,24^ (Figs. 2, 3, 5), their primary subcellular localization differs. Since their individual protein complexes may share some identical components (Fig. 8), the two cyclophilins may regulate the same substrates in coordination or in competition when they coexist in the same cellular compartment, but they may also exert differential effects on specific substrates in other cellular compartments. As to the structures of the two cyclophilins, *Tv*CyP1 is a homodimer^45^, whereas *Tv*CyP2 is a monomer like *h*CyPA (Chen CP, personal communication). Being dimeric renders *Tv*CyP1 capable of binding to either a single substrate with two Gly-Pro dipeptide motifs or to two copies of the same or distinct substrates, each with a single dipeptide motif. Given that the two cyclophilins may also coexist in the same protein complex, with direct interactions in a reciprocal enzyme-substrate relationship, the complexity of cyclophilin-mediated functional regulation can be greatly expanded and very difficult to decipher. The amino acid at Ser^123^ in the conserved enzyme pocket of *Tv*CyP2 is of particular interest (Fig. 1B). In other *h*CyPA homologues, the corresponding amino acid is Ala, and β-CH3 strongly interacts with ηCH3 of MeBmt-1, γCH3 of Abu-2, and NCH3 of MetLeu-4 in CsA^46^. As one of the gatekeeping residues, Ser^123^ is a polar amino acid that may affect the strength of the hydrophobic core to interact with CsA or specific substrates. This difference may provide a lead for rational drug design to treat parasitic infections without affecting the human host.

With the disulfide linkages present in Myb3 (S. H. Chen, unpublished data) and detection of Myb3 in membrane fractions enriched in ERs or Golgi complexes (Fig. 4H), Myb3 may mature in the ER, where the high oxidative potential favors the formation of disulfide linkages^47^. It is possible that initial interactions among *Tv*CyP2, *Tv*CyP1, and Myb3 may occur in the ER. Since *Tv*CyP2 and *Tv*Bip are both chaperones that coexist with *Tv*CyP1 and Myb3 in the same protein complexes (Fig. 8), they may act in coordination to mediate protein maturation or protein-protein interactions of *Tv*CyP1 and Myb3 in the ER for subsequent trafficking through the endomembrane system. *Tv*CyP1 and *Tv*CyP2 were shown to antagonistically regulate the accumulation of Myb3 in the V_myb_ fraction as well as the nuclear import of Myb3^24^ (Figs. 6, 7C), but they had similar effects on the level of Myb3 in the P100/Myb3IP_hmw_ and P100/Myb1 membrane fractions^24^ (Fig. 7C), implying that cyclophilin-mediated trafficking of Myb3 through different cellular compartments may involve other rate-limiting regulatory proteins like *Tv*HSP72 in the cytosol and Myb3IP_hmw_ in the P100/Myb3IP_hmw_ compartment (Fig. 8). This scenario might be further complicated by the role of *Tv*CyP2 in the subcellular distributions of a few other membrane proteins, such as Myb1, *Tv*CyP1, and Myb3IP_hmw_ (Fig. 7C). Thus, the cellular mechanism underlying the membrane trafficking of Myb3 is far more complicated than our current understanding.

**Figure 10:**
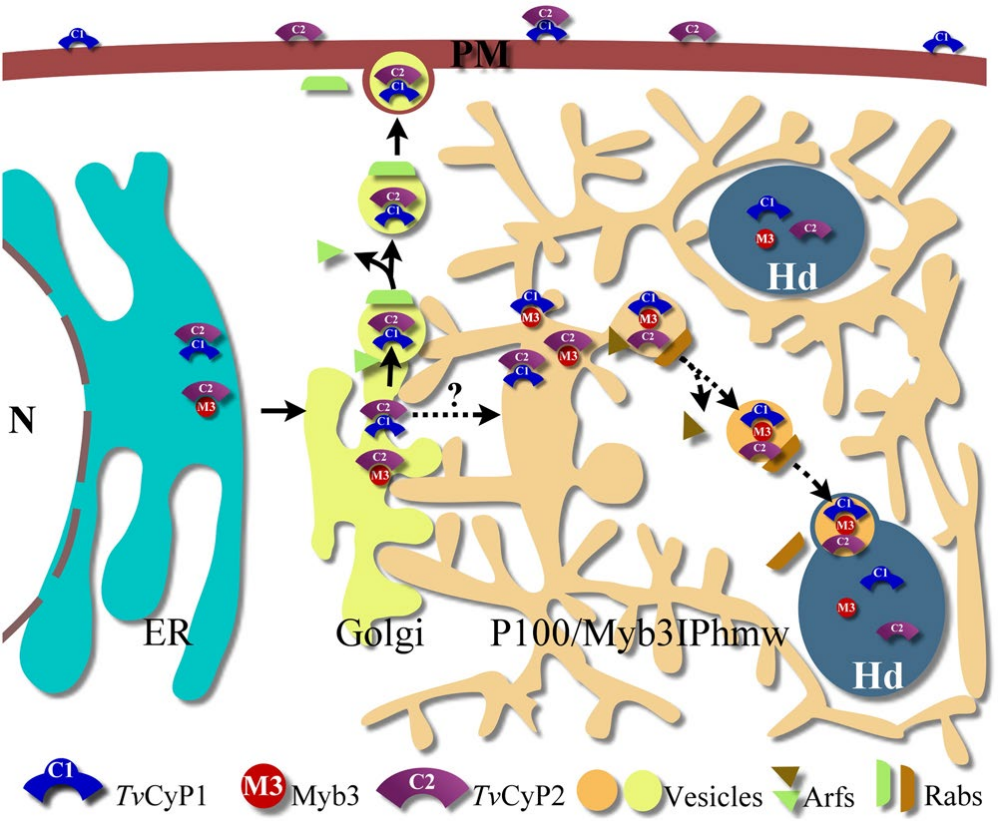
A hypothetical pathway for protein trafficking via the endomembrane system in *T. vaginalis*. With evidence presented herein and elsewhere^24^, a hypothetical pathway for the membrane trafficking of the hydrogenosomal *Tv*CyP1 and Myb3 transcription factor is proposed. In this scheme, *Tv*CyP2, *Tv*CyP1, and Myb3 in the endoplasmic reticulum (ER) along with a *Tv*Bip co-chaperone are assembled into distinct protein complexes, where *cis*-*trans* conformational switches on individual proteins may occur. According to the eukaryotic paradigm^61^, these proteins are likely transported to the Golgi to be further modified and sorted into various vesicles for the delivery to defined membrane compartments. Presumably, a particular Arf protein mediates the budding of distinct vesicles from a specific membrane compartment, whereas a specific Rab protein directs the vesicles to a defined destination. In *T. vaginalius*, an ill-characterized Myb3IP_hmw_ compartment (see Fig. 5A) may serve as an intermediate network to receive the cargos from the Golgi for further processing before they reach the final destinations. In the scheme, the cargos packed with *Tv*CyP1 and *Tv*CyP2 may be delivered to plasma membrane (PM) from the Golgi as indicated by a solid line. An alternative pathway may exist post-Golgi to deliver some of the cargos packed with *Tv*CyP1 and Myb3 to Myb3IP_hmw_ compartment for further processing before transporting to the hydrogenosomes (Hd) as indicated by a dash line.

ER proteins in higher eukaryotes usually contain an N- terminal signal peptide enriched in hydrophobic amino acids as well as an ER retention motif, XDEL or KKXX, at the C- terminus like the human Bip^48,49^. Yet such signals may not be present in *Tv*CyP2 as assessed either by aligning its N- and C-terminal sequences with *Tv*Bip and human Bip (Supplementary Fig. 22) or by an in silico sequence analysis (http://www.cbs.dtu.dk/services/SignalP/). On the other hand, Signal peptides involved in the targeting of certain hydrogenosomal proteins from the cytosol to the hydrogensomes are also absent in *Tv*CyP1 and Myb3 as well as many other hydrogenosomal proteins^50–52^ (Supplementary Fig. 22). In this regard, cryptic signal peptides embedded in the sequences of such hydrogenosomal proteins have been proposed without experimental evidence^52^. Together with the findings in the present report, *Tv*CyP1 and Myb3 are likely transported from ER to the hydrogenosomes via a novel protein trafficking pathway (see Fig. 10).

Current knowledge of the endomembrane system in this primordial unicellular parasite is limited to morphological observations of intracellular organelles, like the ER, Golgi complex, and lysosomes, or putative tubulovesicular transport intermediates, like the V_myb_ and the P100/Myb3IP_hmw_ compartments^18^ (Fig. 5A). Thus, sequential events in the protein trafficking of Myb3 or *Tv*CyP1 via the endomembrane system could not be well defined. Since *Tv*CyP2 is primarily located in the ER, it is likely to be the master regulator of this trafficking pathway. Notably, *Tv*CyP1, as a bona fide hydrogenosomal protein devoid of a typical N-terminal signal peptide^18,51^, was localized on the plasma membrane and in hydrogenosomes (Fig. 7D). The level of *Tv*CyP1 in the two cellular compartments as regulated by the enzymatic proficiency of *Tv*CyP2 seemed to be interrelated, in that as more of *Tv*CyP1 localizes in the hydrogenosomes, there is less on the plasma membrane (Fig. 7C,D). Thus, it is plausible that *Tv*CyP1 delivered to the two cellular compartments probably originated from the same pool, most likely the ER. It is tempting to speculate that Myb3 and *Tv*CyP1 mature in the ER and are then transported to the Golgi complex. The P100/Myb3IP_hmw_ compartment may relay *Tv*CyP1 or Myb3 sorted from the Golgi complex delivering to the V_myb_, hydrogenosomes and plasma membrane through the regulation of conformational switching catalyzed by *Tv*CyP2 (Fig. 10). Future studies on the functional characterization of the P100/Myb3IP_hmw_ and V_myb_ compartments are important to prove or disprove this hypothesis. Nonetheless, like the eukaryotic paradigm for protein trafficking in the endomembrane system, the pathway identified herein may also involve specific Arf-like and Rab-like proteins for vesicular transport and the fusion of vesicles with distinct membrane compartments^53^. These speculations are supported by the morphological evidence on the fusion of vesicles with hydrogenosomes (Fig. 9A). The tools and information described herein will be useful for identifying and characterizing how Arf and Rab are specifically involved in this intriguing protein trafficking pathway (H. M. Hsu, C. H. Chu, and J. H. Tai, unpublished data). Nonetheless, this novel pathway is distinct from that of a typical hydrogenosomal protein with an N-terminal signal peptide^50–52^, which is imported from the cytosol into hydrogenosomes via a system similar to that of the mitochondrial Tim/Tom translocase system^54,55^.

In summary, our observations suggest that a novel protein trafficking pathway from the ER en route to hydrogenosomes and the plasma membrane may exist in this primordial eukaryote. Since the deleterious outcomes of trichomoniasis are probably caused by a number of secretory proteins, including many proteases, a macrophage migration inhibition factor-like protein, and those in the exosomes^56–60^, our study may also provide a handle for further study of the ill-defined secretory pathway for these virulence factors.

## Supplementary information

Supplementary information.

## Data Availability

All data generated or analysed during this study are included in this published article (and its Supplementary Information files)^61^.
