# Supplementary material for: Endomembrane Protein Trafficking Regulated by a TvCyP2 Cyclophilin in the Protozoan Parasite, Trichomonas vaginalis
Source: Sci Rep. 2020 Jan 27;10:1275. doi: 10.1038/s41598-020-58270-6 (PMC6985235; doi:10.1038/s41598-020-58270-6)
Supplement: Supplementary file 1 — Supplementary information. [file 41598_2020_58270_MOESM1_ESM.pdf]

Supplementary Information

Endomembrane Protein Trafficking Regulated by a *Tv*CyP2 Cyclophilin in the Protozoan Parasite, *Trichomonas vaginalis*

Hong-Ming Hsu[1], Yu-Hsin Huang[2], Sarita Aryal[3], Hsing-Wei Liu[2], Chinpan Chen[3] Shu-Hui Chen[4], Chien-Hsin Chu[2,*], and Jung-Hsiang Tai[1,2,*]

From the [1]Department of Tropical Medicine and Parasitology, College of Medicine, National Taiwan University, Taipei, Taiwan; Divisions of [2]Infectious Diseases and Immunology and [3]Structural Biology, Institute of Biomedical Sciences, Academia Sinica, Taipei, Taiwan; [4]Department of Chemistry, National Cheng Kung University, Tainan, Taiwan.

[*]Address correspondence to:

Jung-Hsiang Tai, Division of Infectious Diseases and Immunology, Institute of Biomedical Sciences, Academia Sinica, Taipei 11529, Taiwan, Tel: 886-2-26523934; Fax: 886-2-27858847; E-Mail: taijh@gate.sinica.edu.tw

Chien-Hsin Chu, Division of Infectious Diseases and Immunology, Institute of Biomedical Sciences, Academia Sinica, Taipei 11529, Taiwan, Tel: 886-2-27899163; Fax: 886-2-27858847; E-Mail: therion@gate.sinica.edu.tw

Supplementary Figure 1

Fig. 3B

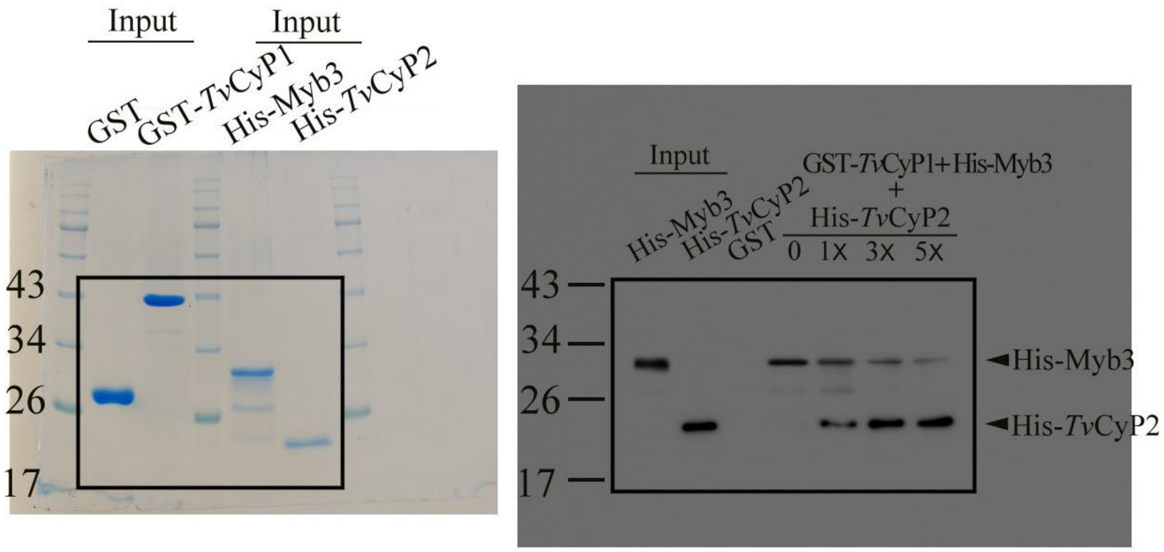

Supplementary Figure 1. Raw data from the Coomassie blue gel staining and Western blotting in Figure 3B. The boxed regions are shown in the manuscript. See figure legends in the manuscript for description.

Supplementary Figure 2

Fig. 3C

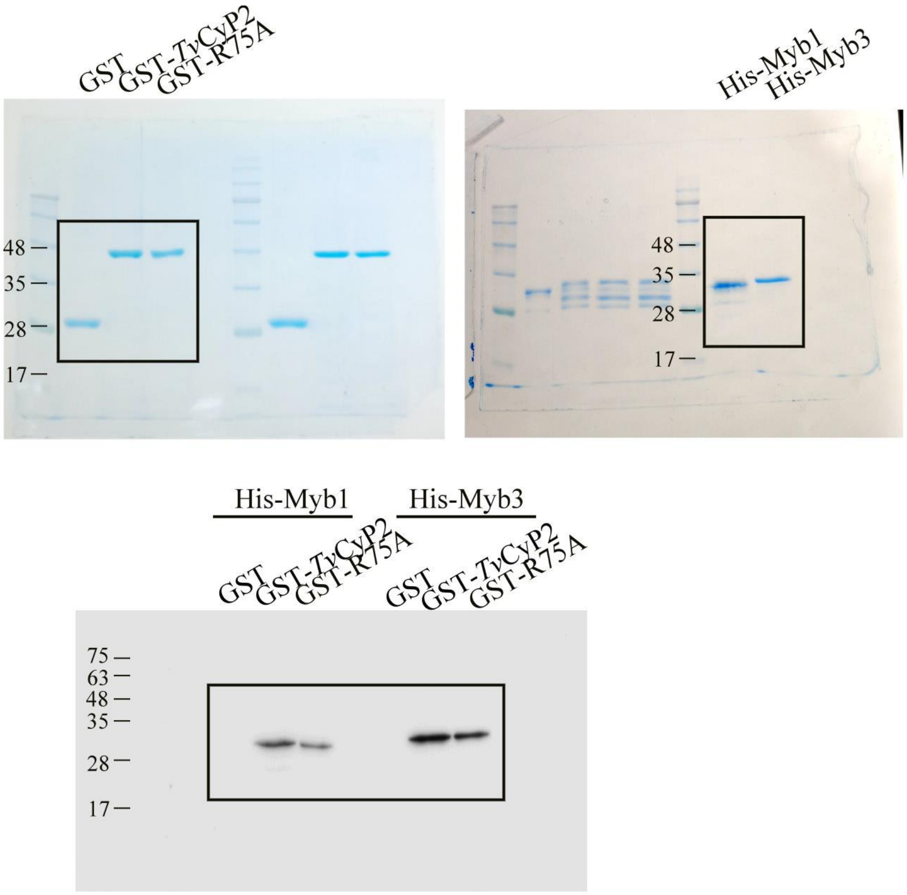

Supplementary Figure 2. Raw data from the Coomassie blue gel staining and Western blotting in Figure 3C. The boxed regions are shown in the manuscript. See figure legends in the manuscript for description.

Supplementary Figure 3

Fig. 3D

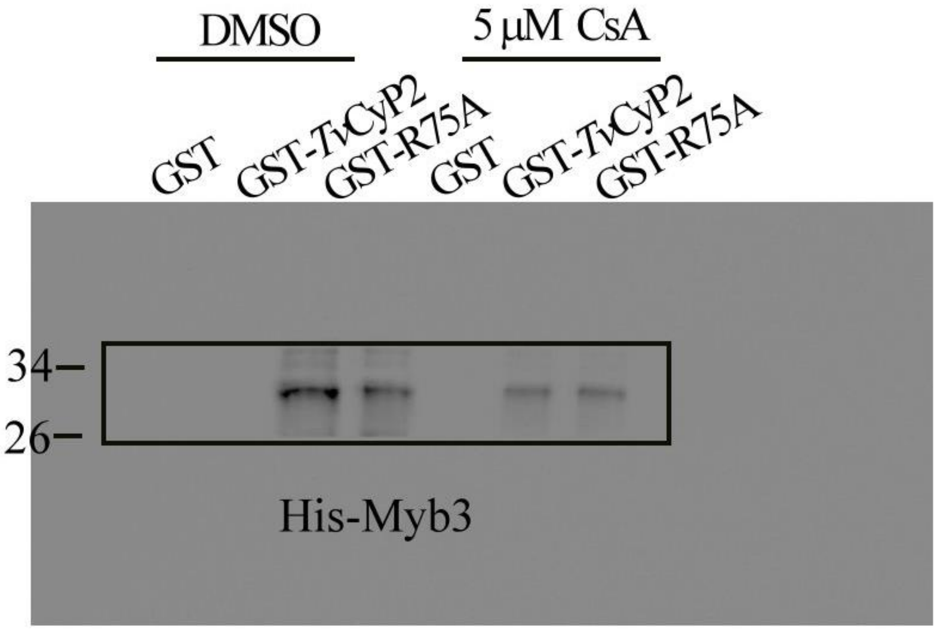

Supplementary Figure 3. Raw data from the Western blotting in Figure 3D. The original membrane blot was sliced into pieces as indicated by the molecular weight markers. Boxed regions are shown in the manuscript. See figure legends in the manuscript for description.

Supplementary Figure 4

Fig. 3E

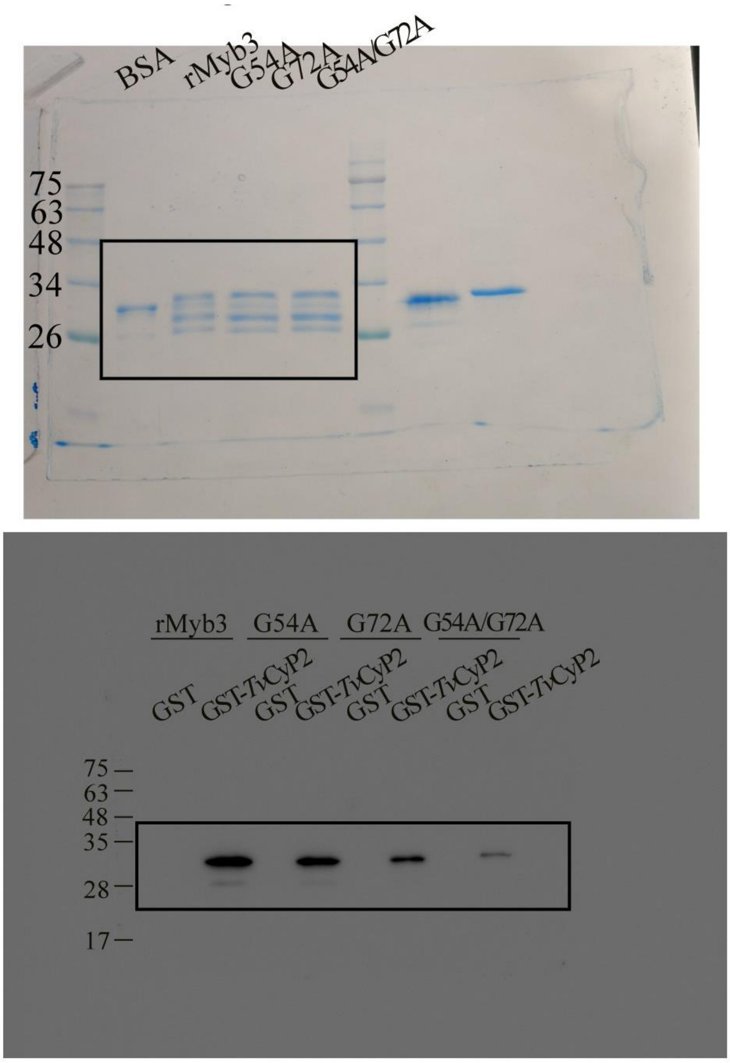

Supplementary Figure 4. Raw data from Coomassie blue gel staining and the Western blotting in Figure 3E. Boxed regions are shown in the manuscript. See figure legends in the manuscript for description.

Supplementary Figure 5

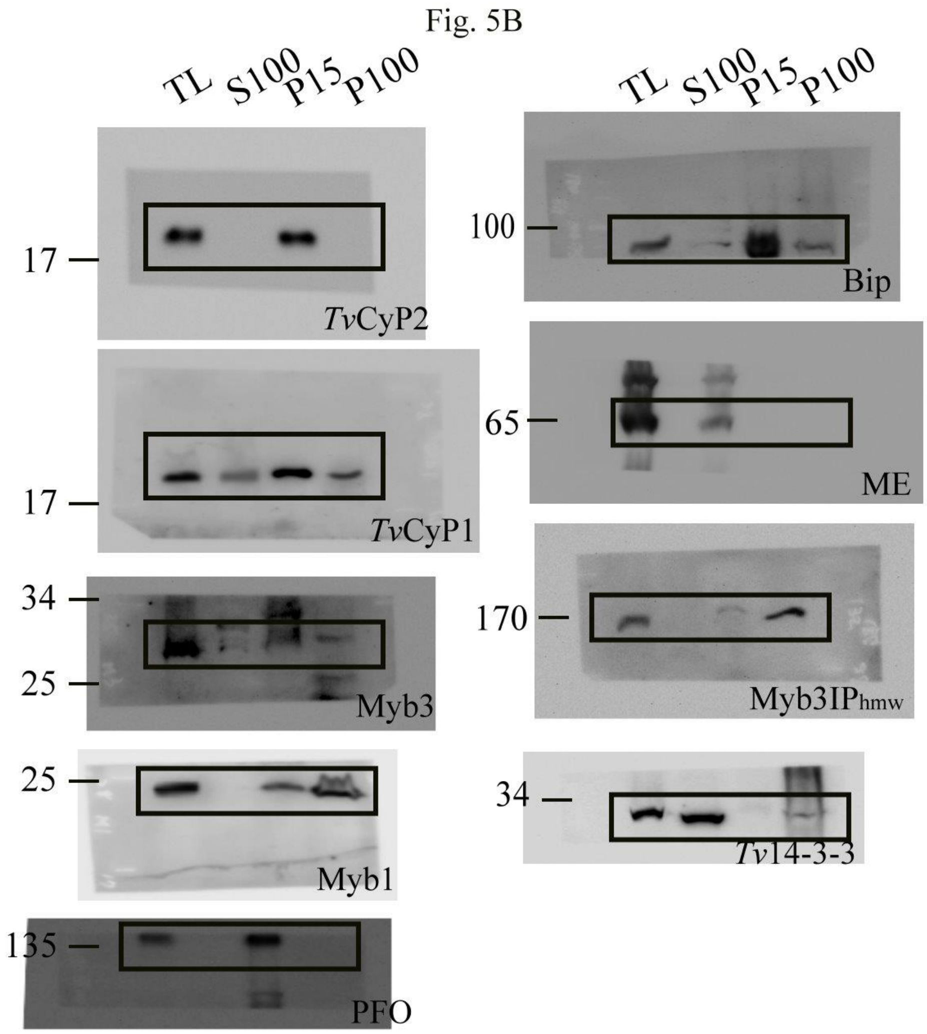

Supplementary Figure 5. Raw data from the Western blotting in Figure 5B. The original membrane blot was sliced into pieces as indicated by the molecular weight markers. Boxed regions are shown in the manuscript. See figure legends in the manuscript for description.

Supplementary Figure 6

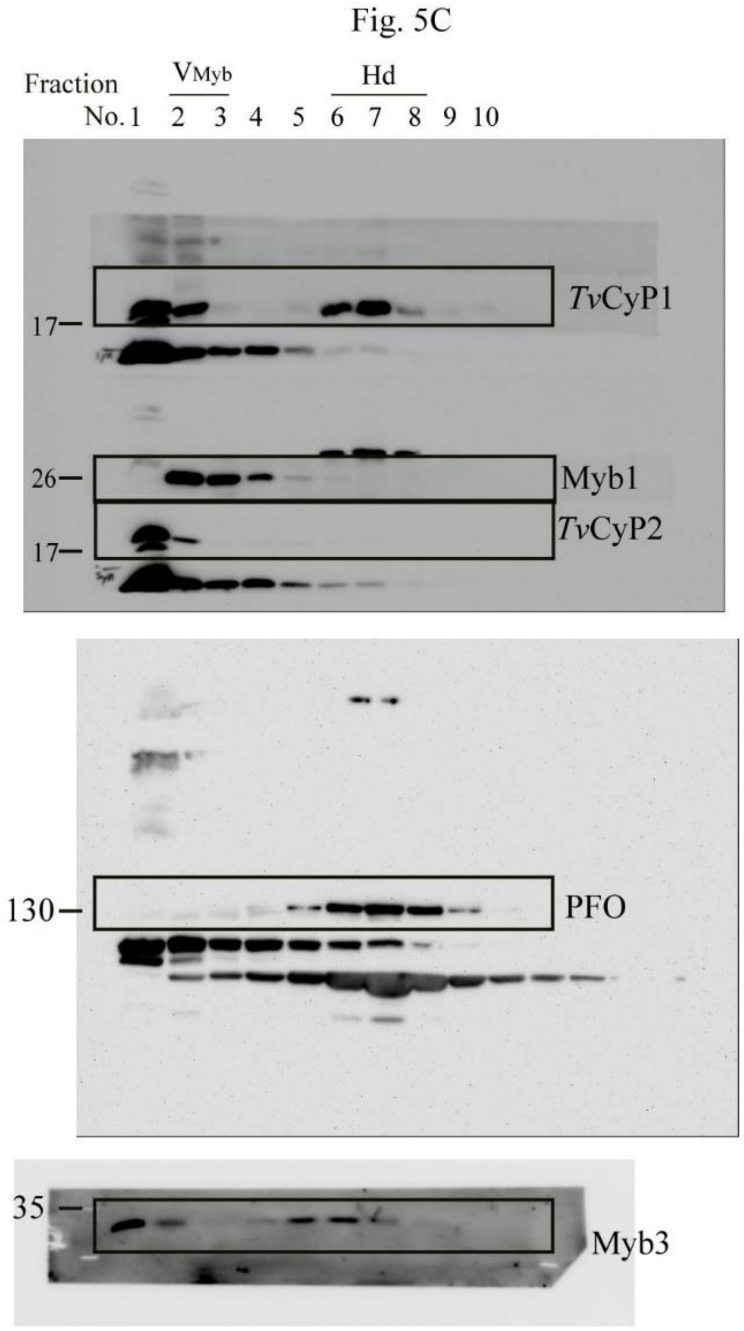

Supplementary Figure 6. Raw data from the Western blotting in Figure 5C. The original membrane blot was sliced into pieces as indicated by the molecular weight markers. Boxed regions are shown in the manuscript. See figure legends in the manuscript for description.

Supplementary Figure 7

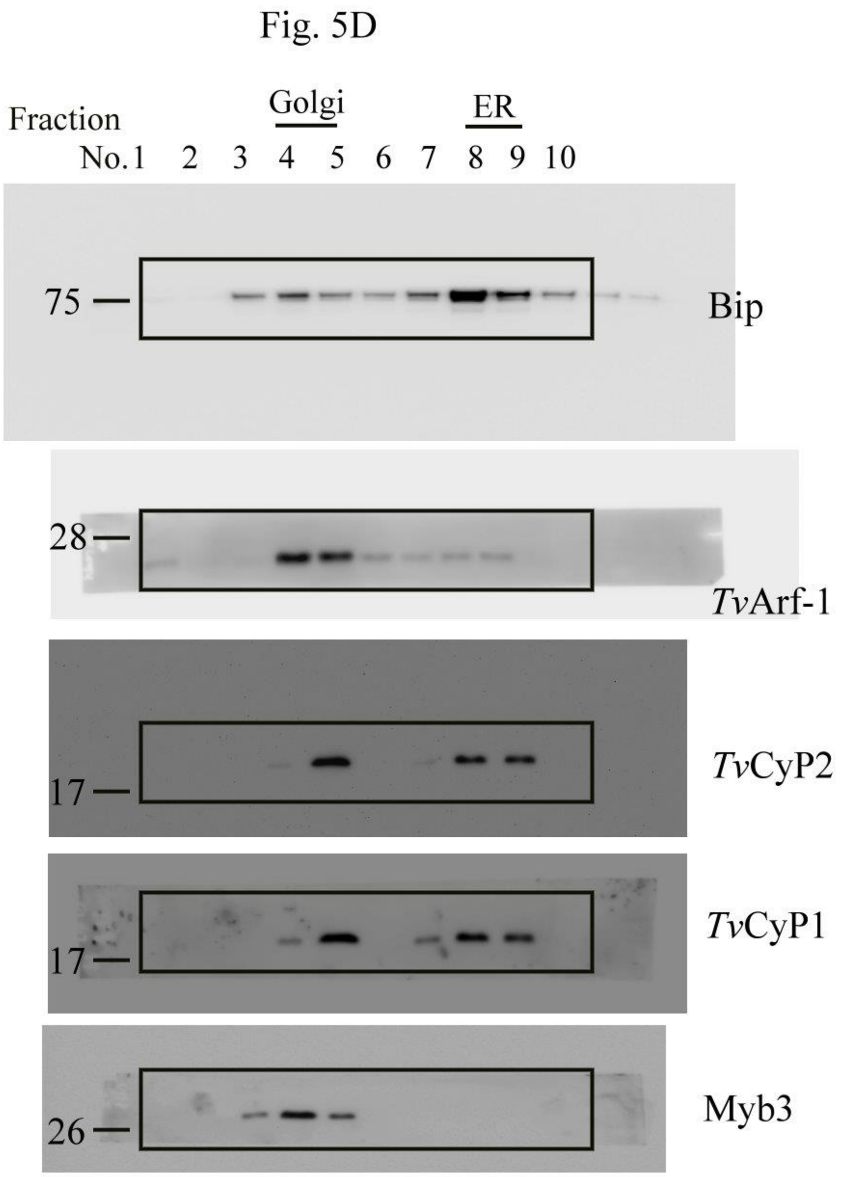

Supplementary Figure 7. Raw data from the Western blotting in Figure 5D. The original membrane blot was sliced into pieces as indicated by the molecular weight markers. Boxed regions are shown in the manuscript. See figure legends in the manuscript for description.

Supplementary Figure 8

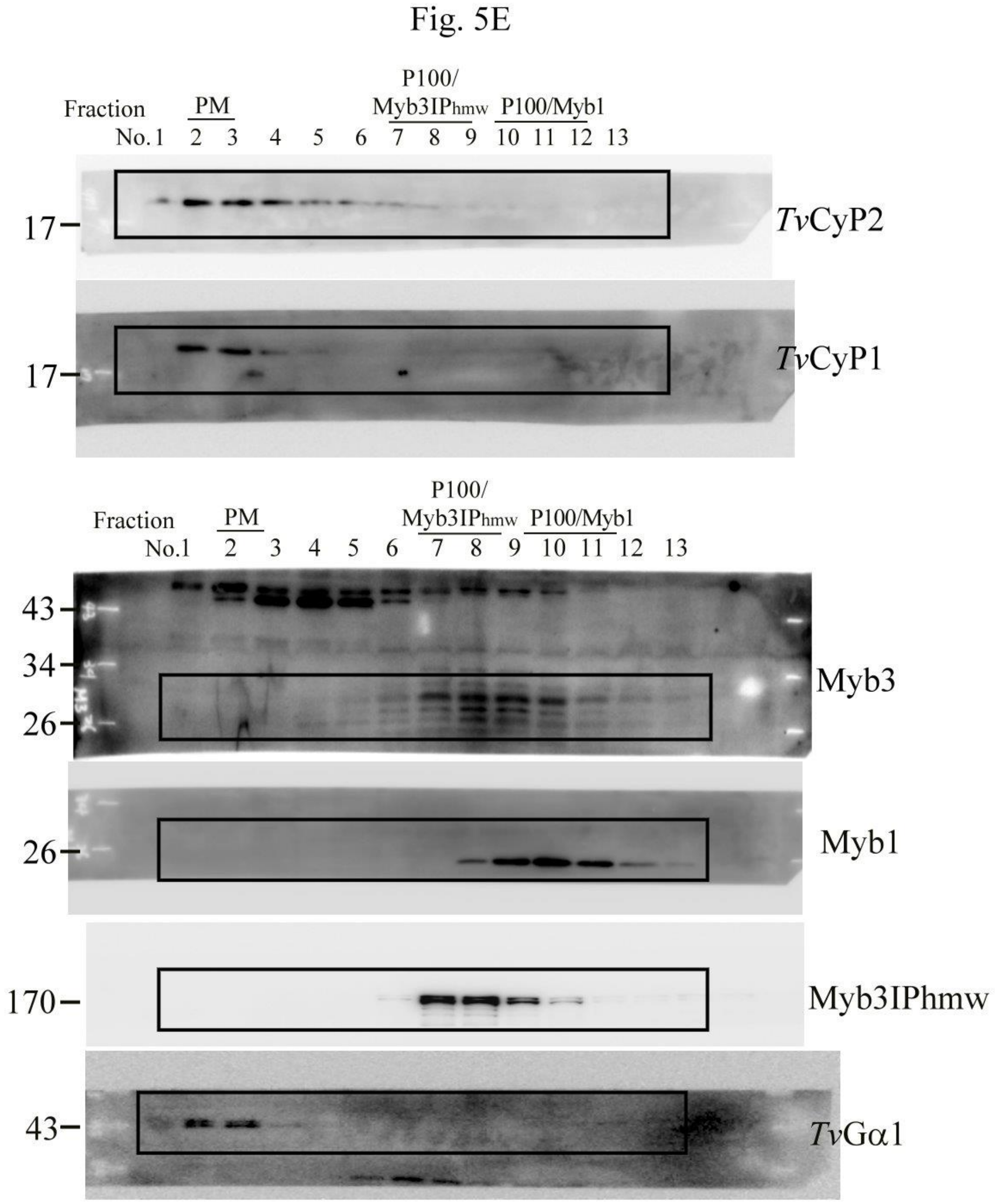

Supplementary Figure 8. Raw data from the Western blotting in Fig. 5E. The original membrane blot was sliced into pieces as indicated by the molecular weight markers. Boxed regions are shown in the manuscript. See figure legends in the manuscript for description.

Supplementary Figure 9

Fig. 6C

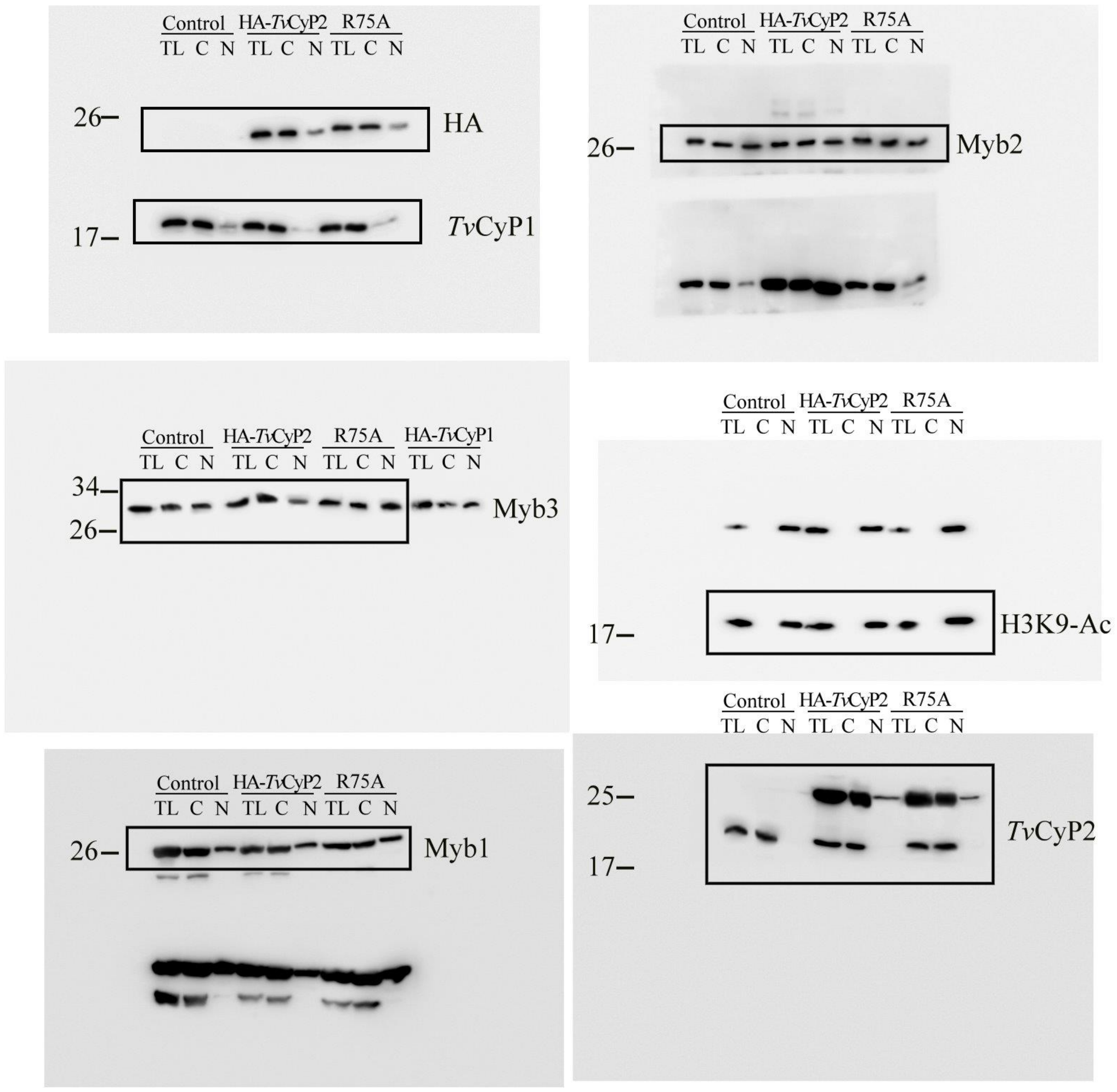

Supplementary Figure 9. Raw data from the Western blotting in Figure 6C. The original membrane blot was sliced into pieces as indicated by the molecular weight markers. Boxed regions are shown in the manuscript. See figure legends in the manuscript for description.

Supplementary Figure 10

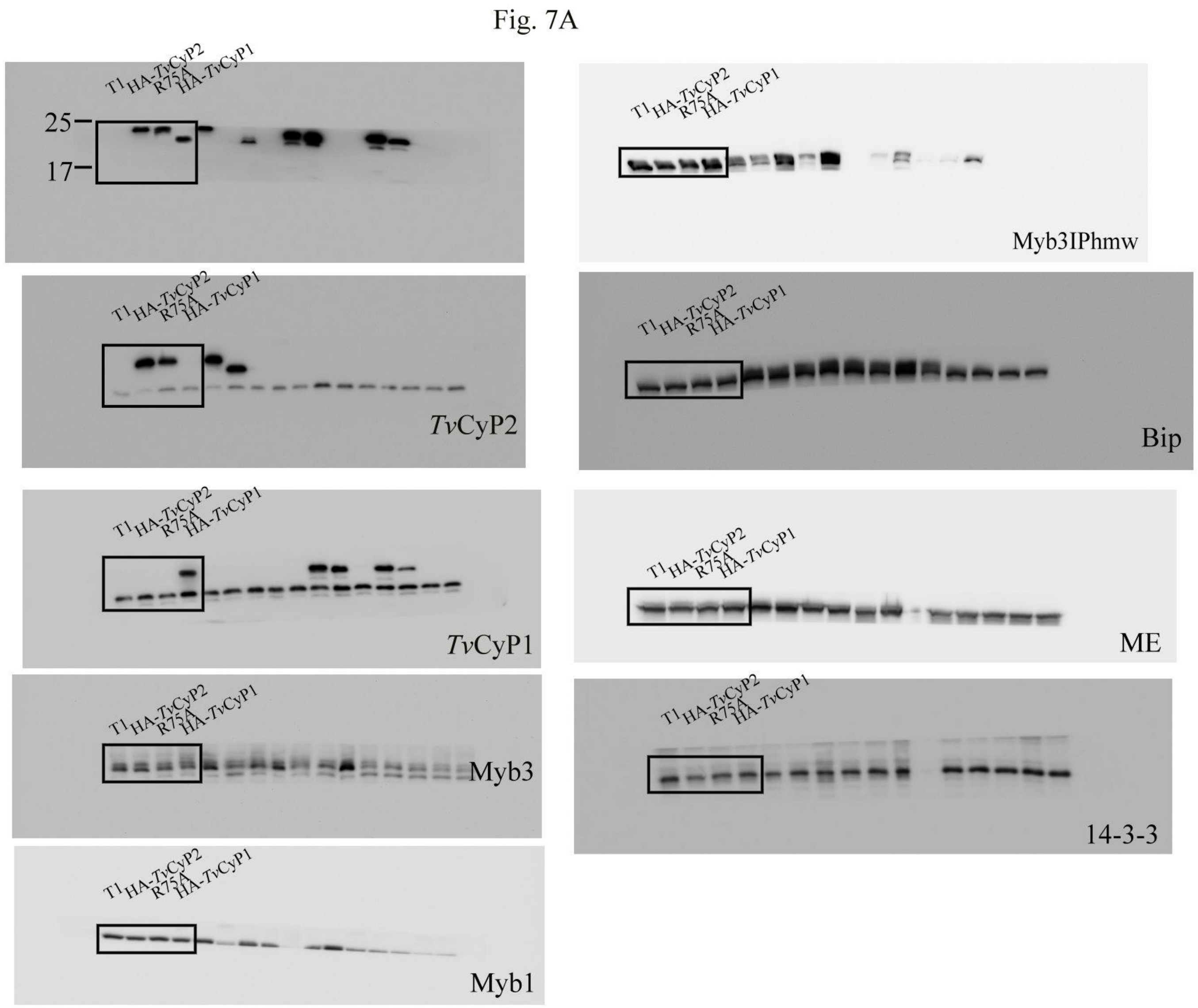

Supplementary Figure 10. Raw data from the Western blotting in Figure 7A. The original membrane blot was sliced into pieces as indicated by the molecular weight markers. Boxed regions are shown in the manuscript. See figure legends in the manuscript for description.

Supplementary Figure 11

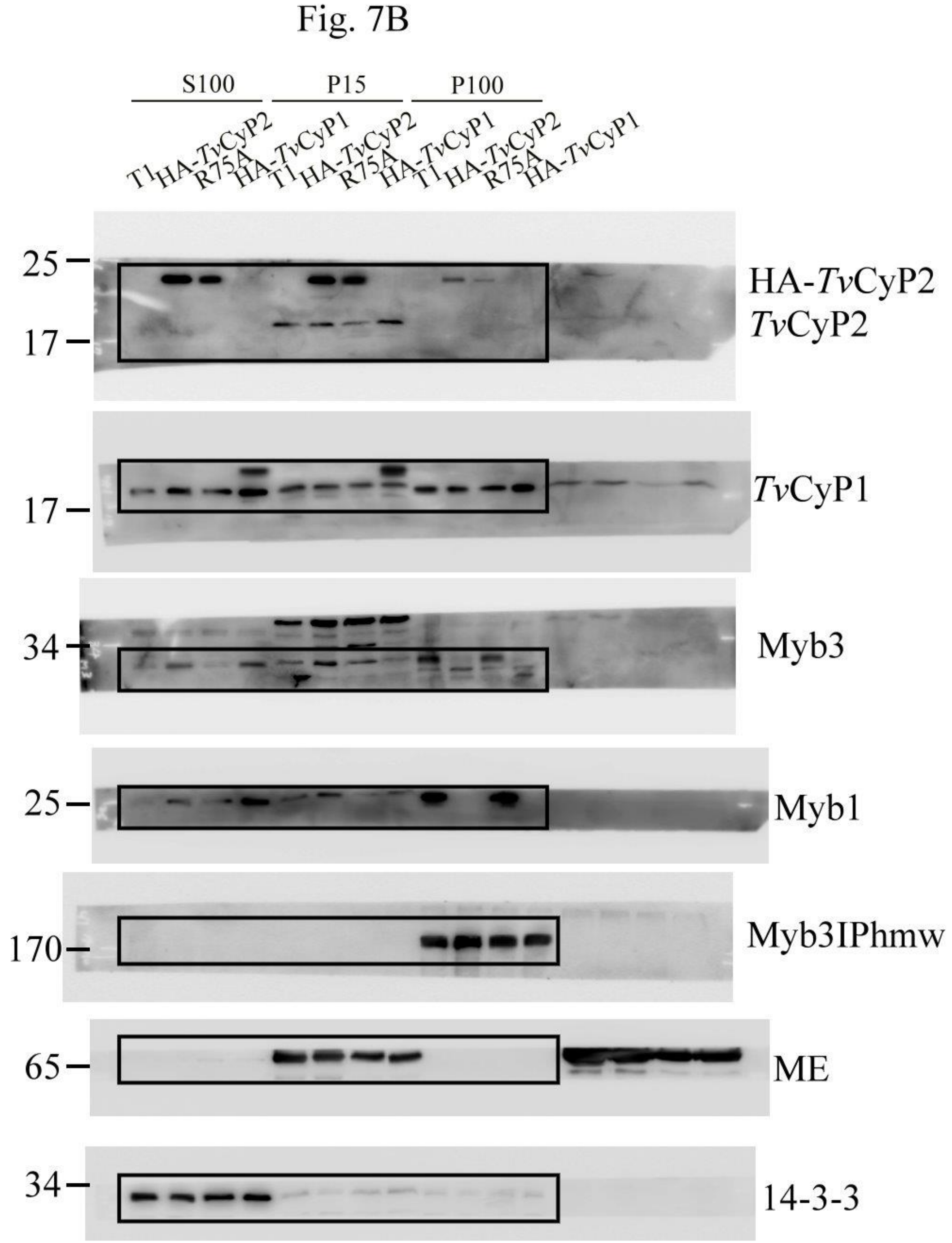

Supplementary Figure 11. Raw data from the Western blot in Figure 7B. The original membrane blot was sliced into pieces as indicated by the molecular weight markers. Boxed regions are shown in the manuscript. See figure legends in the manuscript for description.

Supplementary Figure 12

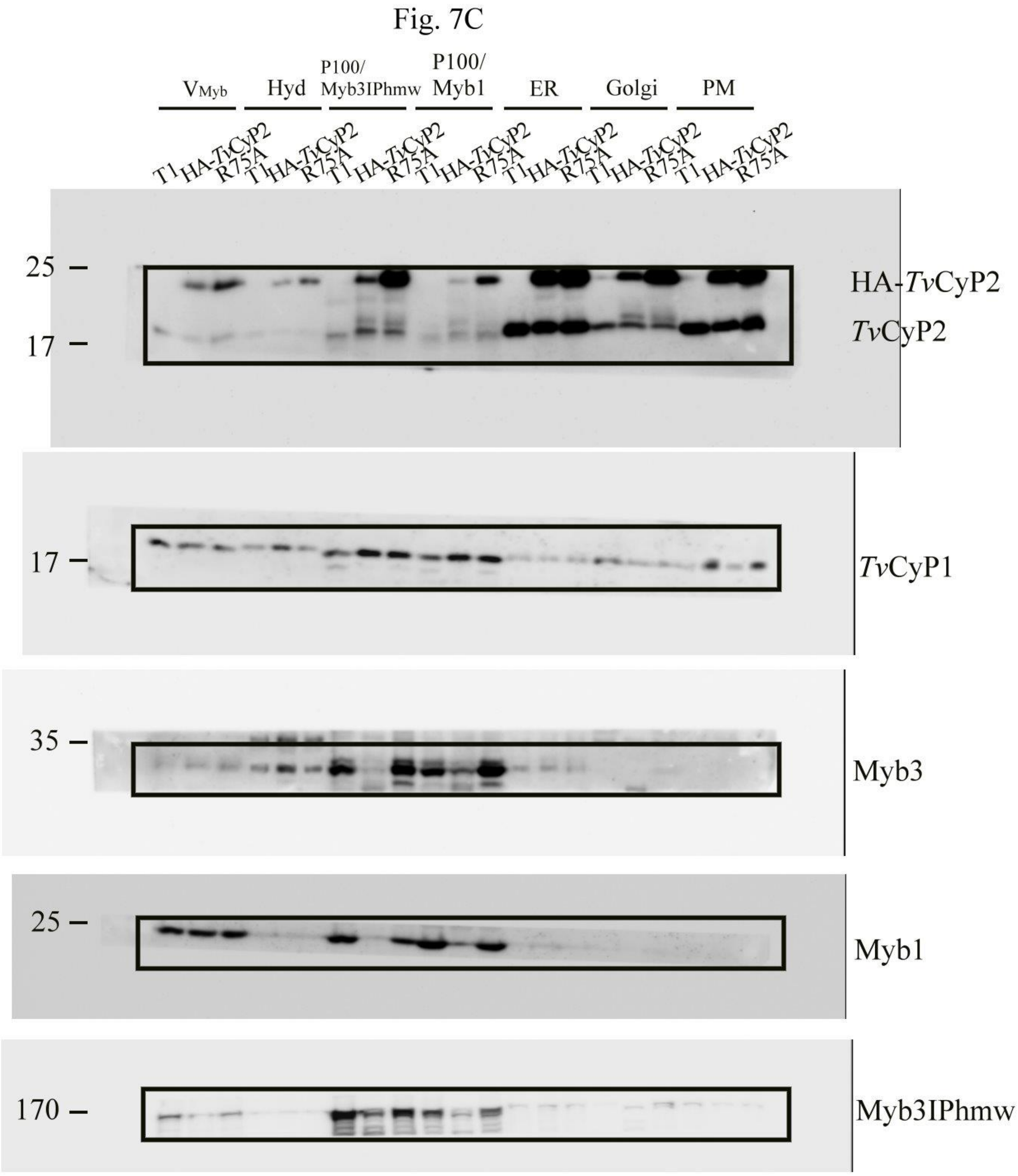

Supplementary Figure 12. Raw data from the Western blotting in Figure 7C. The original membrane blot was sliced into pieces as indicated by the molecular weight markers. Boxed regions are shown in the manuscript. See figure legends in the manuscript for description.

Supplementary Figure 13

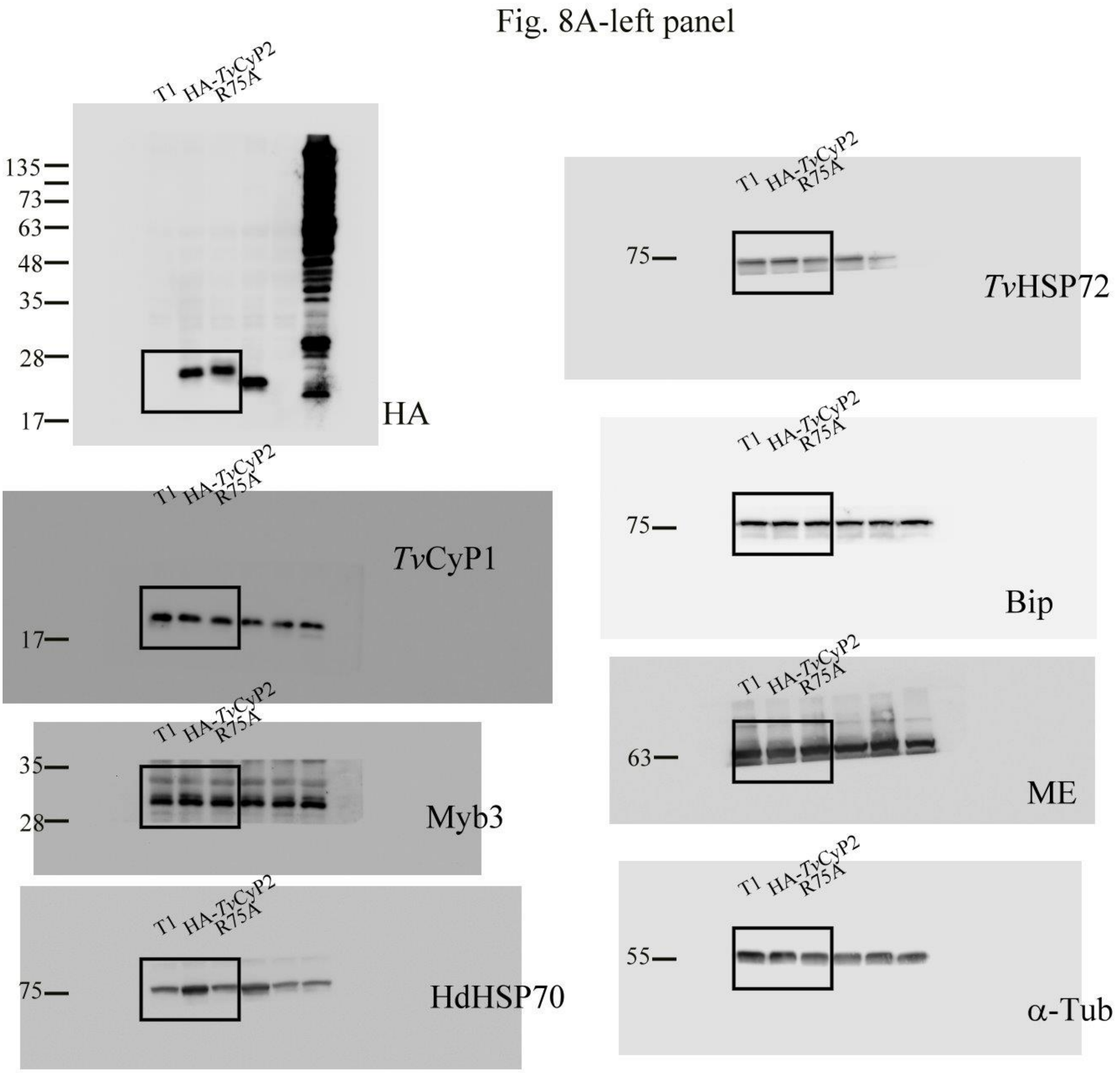

Supplementary Figure 13. Raw data from the Western blotting in the left panel of Figure 8A. The original membrane blot was sliced into pieces as indicated by the molecular weight markers. Boxed regions are shown in the manuscript. See figure legends in the manuscript for description.

Supplementary Figure 14

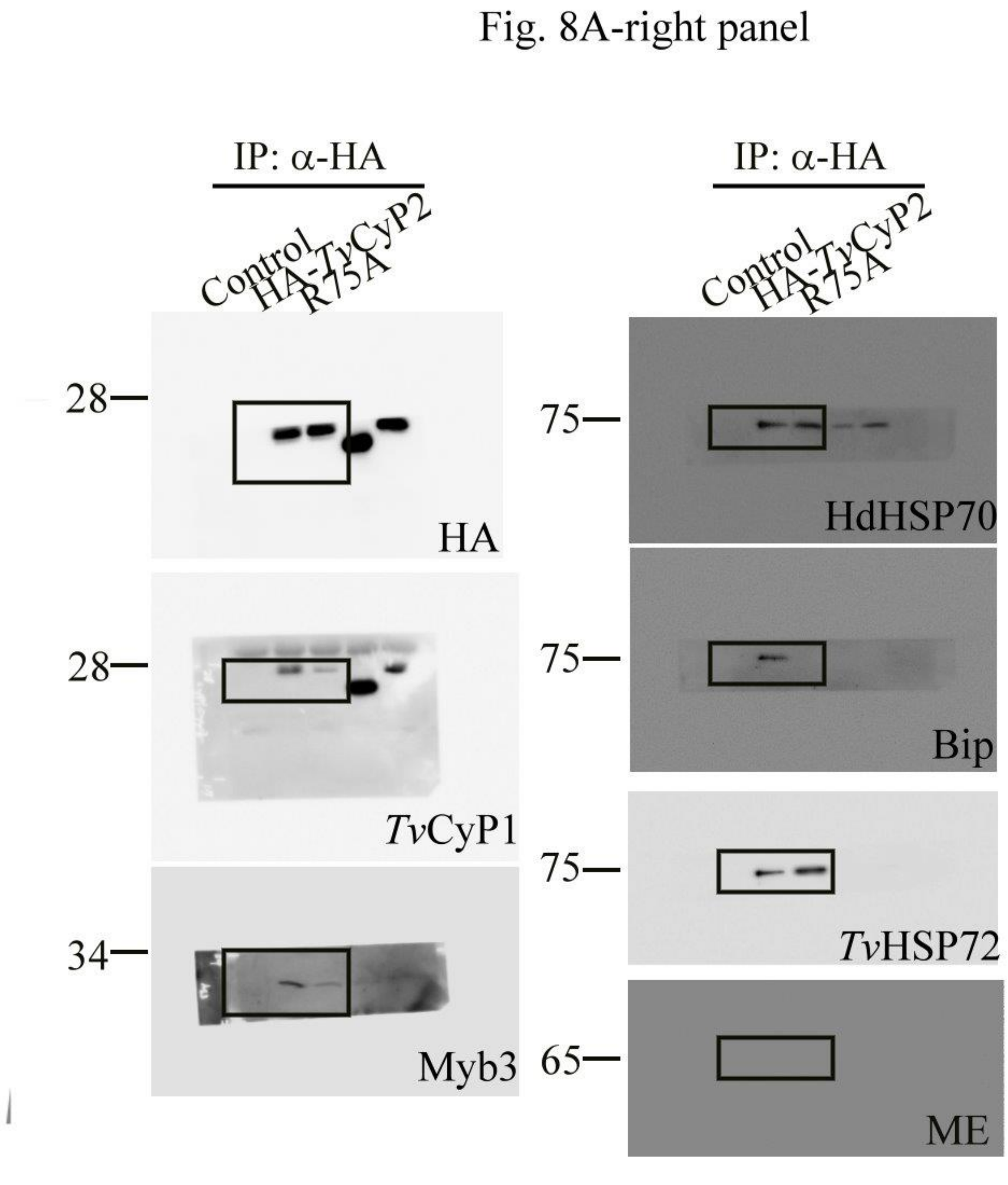

Supplementary Figure 14. Raw data from the Western blotting in the right panel of Figure 8A. The original membrane blot was sliced into pieces as indicated by the molecular weight markers. Boxed regions are shown in the manuscript. See figure legends in the manuscript for description.

Supplementary Figure 15

Fig. 8B and D

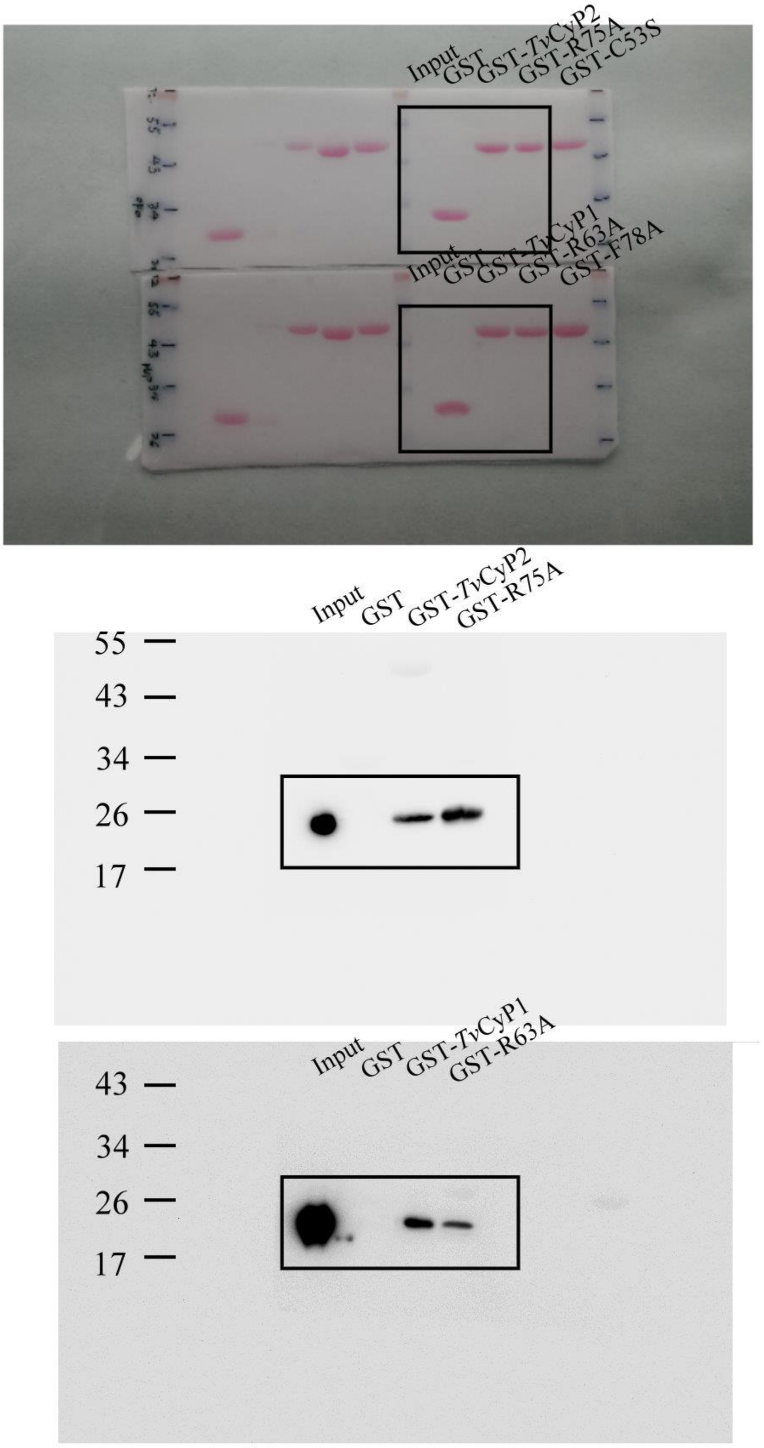

Supplementary Figure 15. Raw data from the Ponceau S staining and Western blotting in Figure 8B and 8D. The original membrane blot was sliced into pieces as indicated by the molecular weight markers. Boxed regions are shown in the manuscript. See figure legends in the manuscript for description.

Supplementary Figure 16

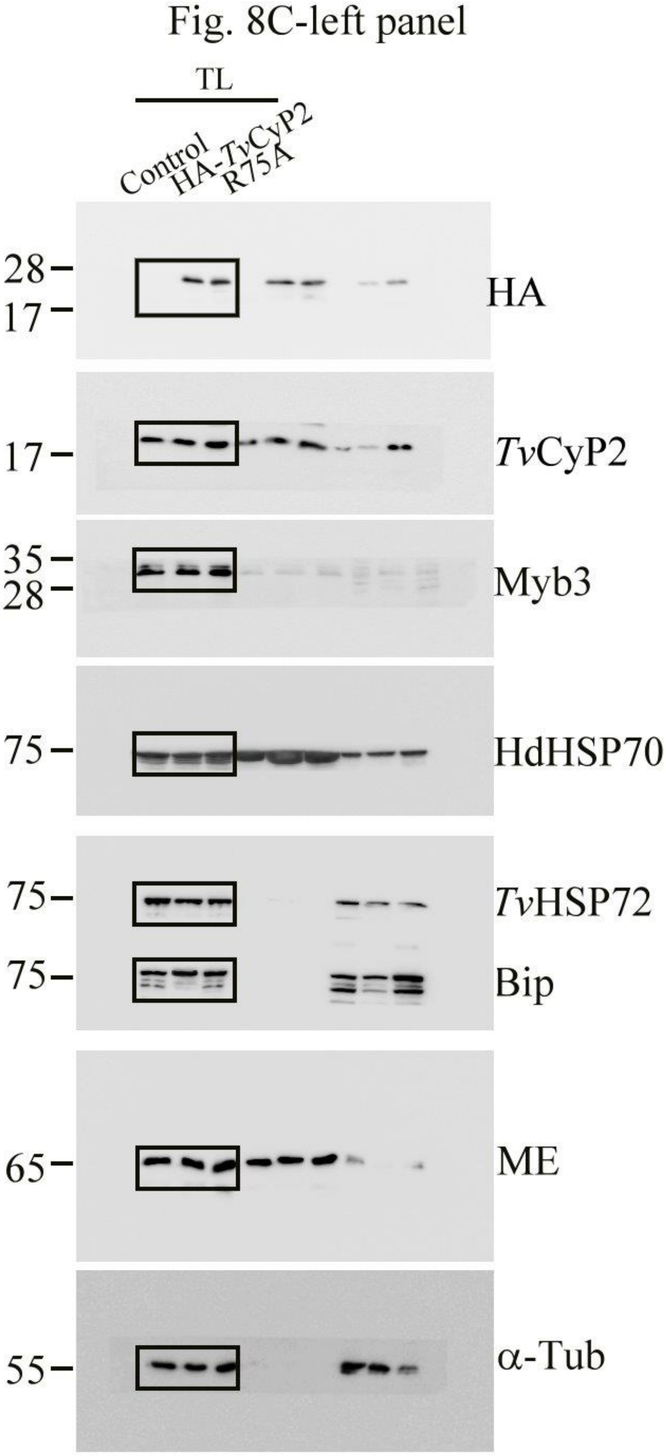

Supplementary Figure 16. Raw data from the Western blotting in the left panel of Figure 8C. The original membrane blot was sliced into pieces as indicated by the molecular weight markers. Boxed regions are shown in the manuscript. See figure legends in the manuscript for description.

Supplementary Figure 17

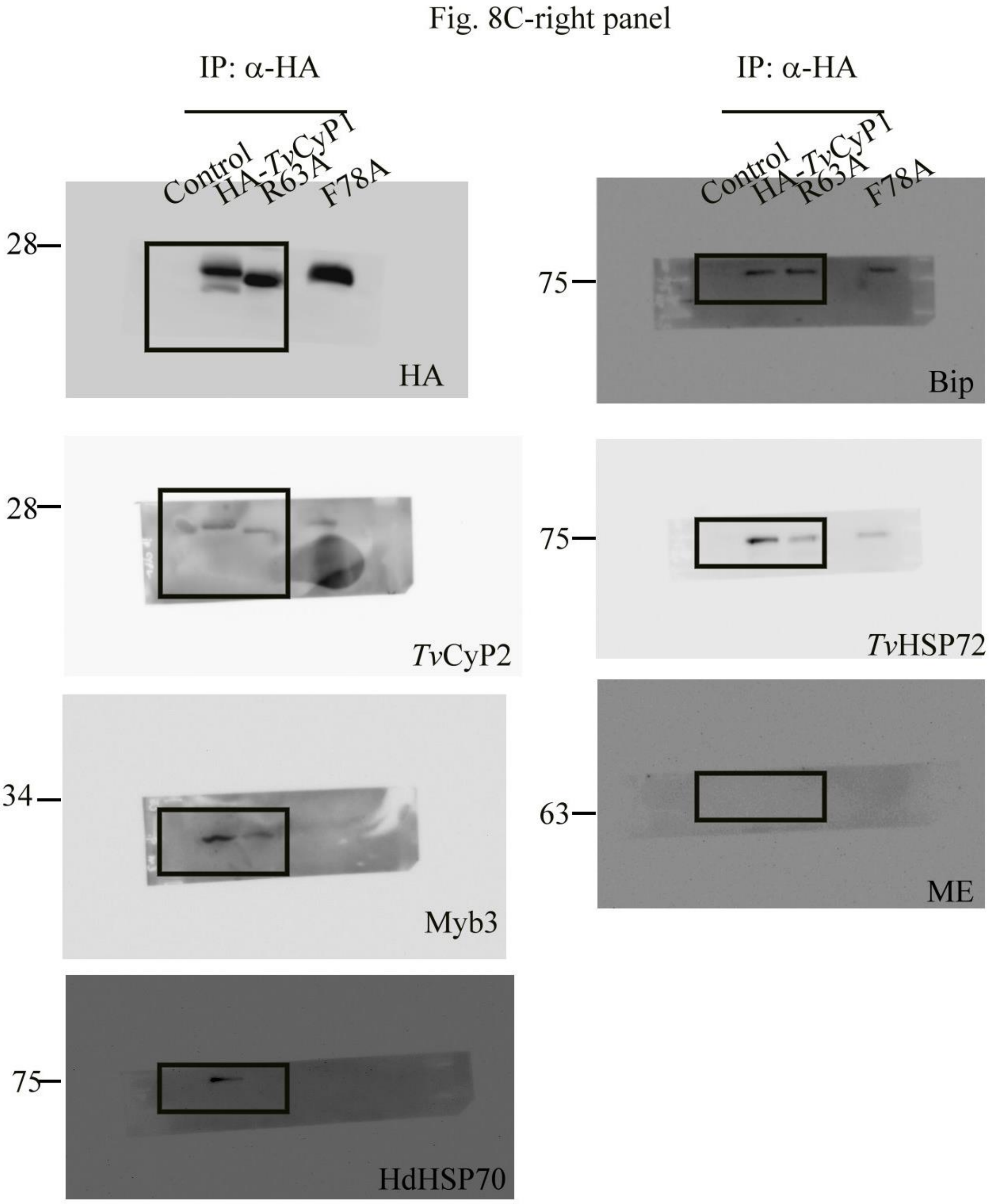

Supplementary Figure 17. Raw data from the Western blotting in the right panel of Figure 8C. The original membrane blot was sliced into pieces as indicated by the molecular weight markers. Boxed regions are shown in the manuscript. See figure legends in the manuscript for description.

Supplementary Figure 18

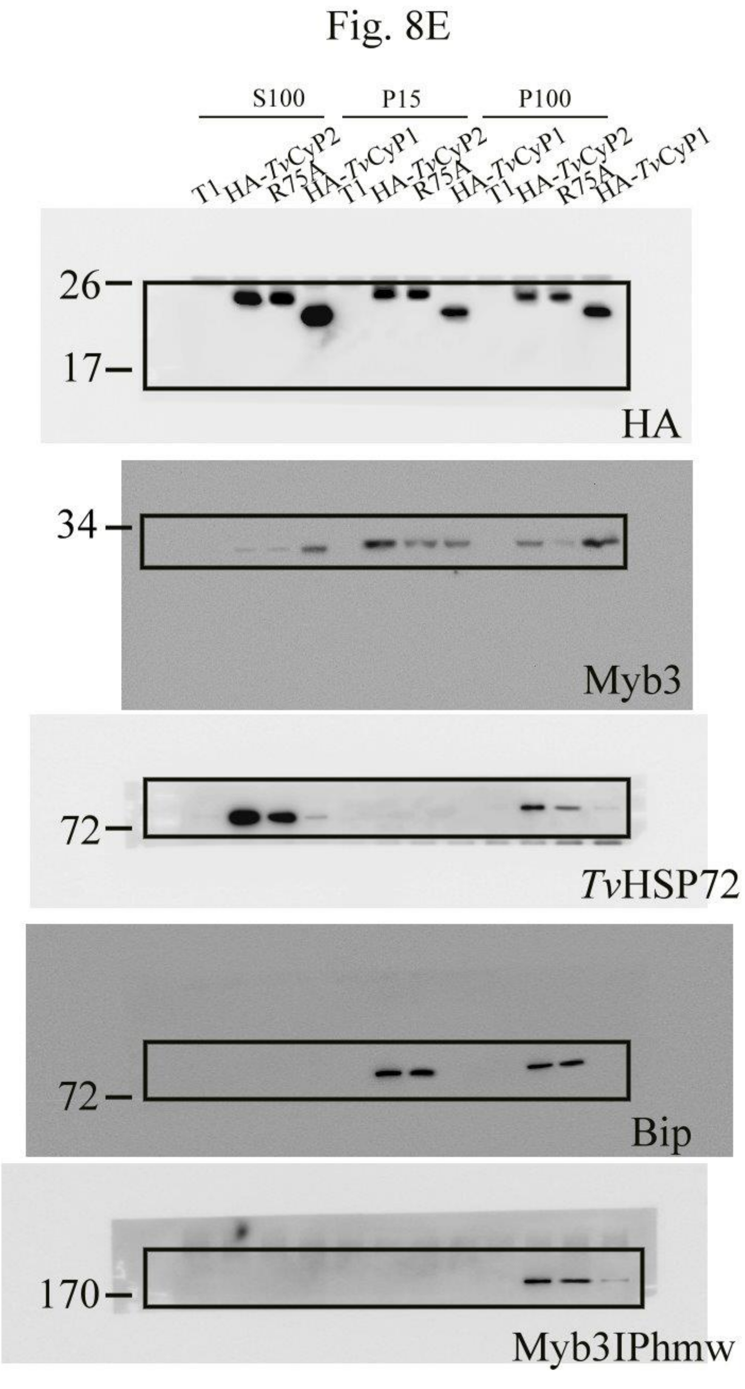

Supplementary Figure 18. Raw data from the Western blotting in Figure 8E. The original membrane blot was sliced into pieces as indicated by the molecular weight markers. Boxed regions are shown in the manuscript. See figure legends in the manuscript for description.

Supplementary Figure 19

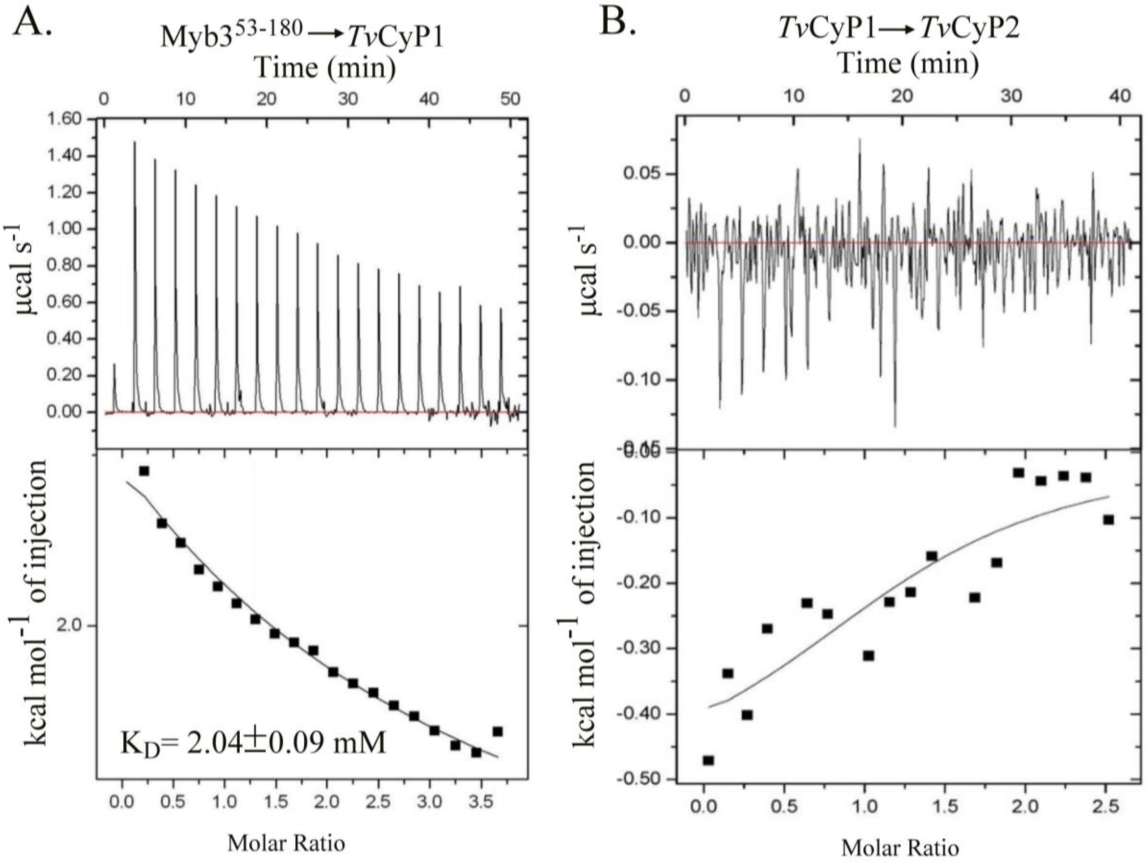

Supplementary Figure 19. The protein interaction measured by ITC. In A, 150 μM of His-*Tv*CyP1 was titrated with increasing amounts of Myb3$^{53\text{-}180}$ (1- 20 μM). In B, 180 μM of His-*Tv*CyP1 was titrated with increasing amounts of His-*Tv*CyP2 (18- 360 μM). ITC was performed at 25°C and K_D value with the standard deviation was measured as shown in each panel.

Supplementary Figure 20

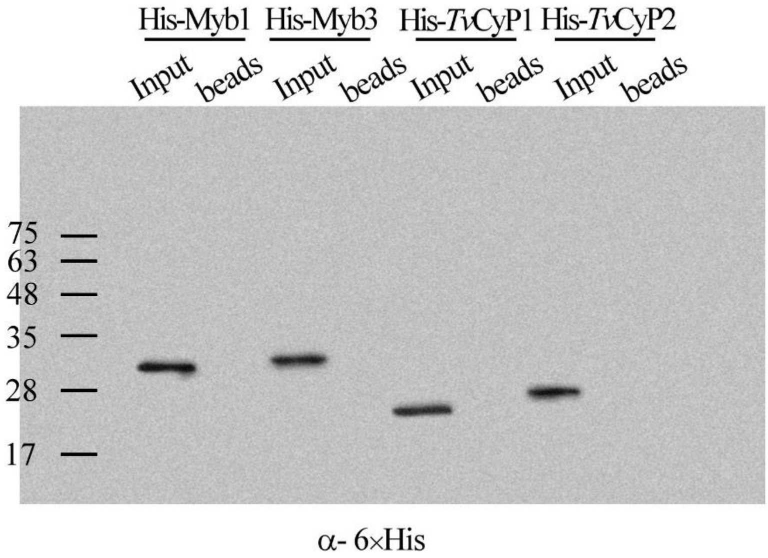

Supplementary Figure 20. Binding specificity of the His-proteins to the glutathione-conjugated beads. Equivalent amount of His-Myb1, His-Myb3, His-*Tv*CyP1, or His-*Tv*CyP2 was incubated with glutathione-conjugated beads for the GST pulldown assay. The pulled down products were analyzed by the Western blotting using the anti-6×His antibody.

Supplementary Figure 21

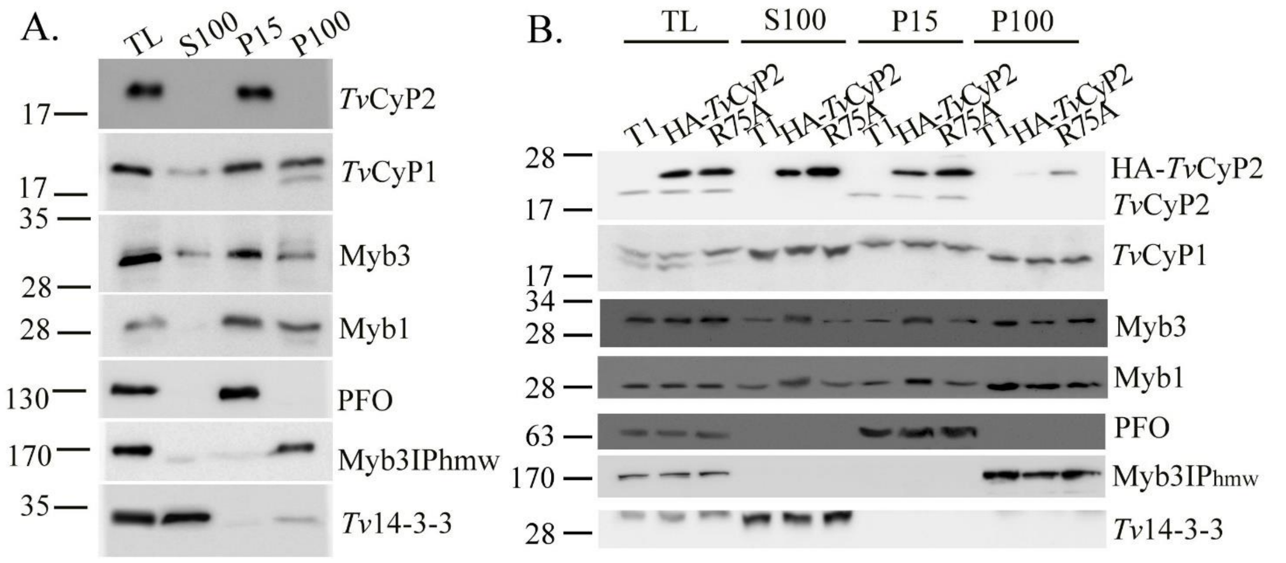

Supplementary Figure 21. Subcellular localization of *Tv*CyP2. The experiments described in Fig. 5B and Fig. 7B of the original manuscript were repeated, and the results shown here are used in the revised manuscript.

Supplementary Figure 22

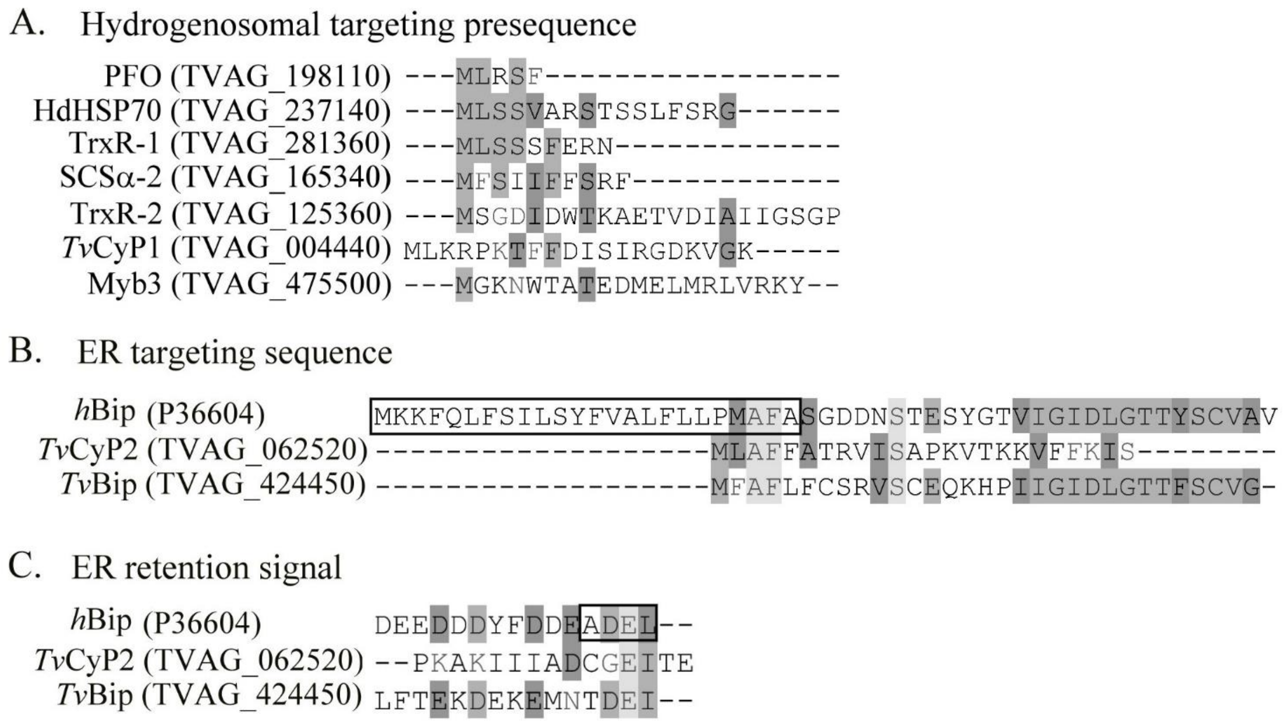

Supplementary Figure 22. Signal peptides in the hydrogenosomal or ER proteins. In A, the N-terminal sequences of several hydrogenosomal proteins, including the pyruvate ferrodoxin oxidoreductase (PFO) (TVAG_198110), HdHSP70 (TVAG_237140), thioredoxin reductase 1 (TrxR-1, TVAG_281360), succinyl CoA synthetase alpha subunit 2 (SCSα-2, TVAG_165340), thioredoxin reductase 2 (TrxR-2, TVAG_125360), and *Tv*CyP1 (TVAG_004440) in *T. vaginlais* are aligned. In B and C, the N-terminal (B) or C-terminal (C) sequences of *Tv*CyP2 (TVAG_062520) and *Tv*Bip (TVAG_424450) in *T. vaginalis* and the human *h*Bip (P36604) are aligned. The ER targeting signal at the N-terminus and the retention signal at the C-terminus of *h*Bip are boxed.
